# Empiric recommendations for population disaggregation under different data scenarios

**DOI:** 10.1371/journal.pone.0274504

**Published:** 2022-09-16

**Authors:** Marta Sapena, Marlene Kühnl, Michael Wurm, Jorge E. Patino, Juan C. Duque, Hannes Taubenböck

**Affiliations:** 1 German Aerospace Center (DLR), German Remote Sensing Data Center (DFD), Weßling, Germany; 2 Company for Remote Sensing and Environmental Research (SLU), München, Germany; 3 Research in Spatial Economics (RiSE-Group), Department of Mathematical Sciences, Universidad EAFIT, Medellin, Colombia; 4 Institute for Geography and Geology, Julius-Maximilians-Universität Würzburg, Würzburg, Germany; Tel Aviv University, ISRAEL

## Abstract

High-resolution population mapping is of high relevance for developing and implementing tailored actions in several fields: From decision making in crisis management to urban planning. Earth Observation has considerably contributed to the development of methods for disaggregating population figures with higher resolution data into fine-grained population maps. However, which method is most suitable on the basis of the available data, and how the spatial units and accuracy metrics affect the validation process is not fully known. We aim to provide recommendations to researches that attempt to produce high-resolution population maps using remote sensing and geospatial information in heterogeneous urban landscapes. For this purpose, we performed a comprehensive experimental research on population disaggregation methods with thirty-six different scenarios. We combined five different top-down methods (from basic to complex, i.e., binary and categorical dasymetric, statistical, and binary and categorical hybrid approaches) on different subsets of data with diverse resolutions and degrees of availability (poor, average and rich). Then, the resulting population maps were systematically validated with a two-fold approach using six accuracy metrics. We found that when only using remotely sensed data the combination of statistical and dasymetric methods provide better results, while highly-resolved data require simpler methods. Besides, the use of at least three relative accuracy metrics is highly encouraged since the validation depends on level and method. We also analysed the behaviour of relative errors and how they are affected by the heterogeneity of the urban landscape. We hope that our recommendations save additional efforts and time in future population mapping.

## Introduction

Accurate knowledge on the spatial distribution of population is of great importance. The development and implementation of tailored and targeted measures is especially challenging in dynamic and high dense urban environments, which is why it has become an essential resource in several fields of research [1, 2]. Some examples underlying the need for accurate, spatially highly disaggregated, and temporally highly resolved population information are in the context of development agendas: e.g., in relation to marginal and uncounted populations [3]; supporting climate change urban resilience and reducing vulnerability [4, 5]; assessing sub-regional exposure and vulnerability in disaster risk management [6] and crisis management services [7]; in the management of the COVID-19 pandemic and analysing the accessibility to medical care [8, 9]; as well as in urban planning and transportation models [10, 11].

Traditionally, census and field surveys were the only mean to obtain population data. These sources usually have a low temporal frequency, provide aggregated data at irregular administrative boundaries and are limited or even non-existent in some areas of the word [2]. In terms of boundaries, there is a recent trend to provide population data in regular grids. For instance, the United States is producing multi-temporal Census 1-km^2^-grid datasets that include a wide variety of socioeconomic characteristics [12]. At European level, similar initiatives are being developed. Currently, Eurostat provides 1-km^2^ population grids for two years using aggregation and downscaling methods, and they are intended to produce the next Census as an EU-wide 1-km^2^-grid. Consequently, some European countries are already delivering statistical grid products (e.g., Austria, Netherlands, Norway and Spain) [13]. These new datasets will allow for cross-border and more flexible analyses tailored to current needs, but their availability is still constrained to the Global North.

Nevertheless, in the era of the digital revolution, Earth observation (EO) has considerably contributed with global, up-to-date, objective, detailed and harmonized indicators to the field of demography [e.g., 14, 15]. EO-derived-data have been used in a number of ways: Firstly, due to the increasing popularity of population grids, researchers have proposed several methods to downscale aggregated population counts available from traditional sources at larger regions relying on geospatial auxiliary information (such as built-up areas, height models, land use, etc.) and statistical techniques, the so-called top-down methods. Providing estimations on the distribution of population at a finer grain level [e.g., 16–19]. Secondly, when ground truth population counts are partially available at the local level (such as georeferenced points, houses or microcensus), geospatial auxiliary variables from EO can be used to build statistical models to estimate population in unsampled locations, which is known as bottom-up methods [2, 17, 20, 21]. Third, the remote identification and characterization of populated places can be used to estimate time series of population filling in data gaps between census or as a proxy of population density without the need to rely on official census and surveys, nor constrained by boundaries or time-scales [22–24]. These methods, although less accurate than the others in absolute terms, provide estimates on population density and distribution in regions where censuses are unreliable, infrequent or not conducted at all.

Ideally, the best possible way to produce gridded population maps is collecting georeferenced point population data and counting the number of people in each grid [25]; however, few countries have microdata available to conduct this approach. Since aggregated population data are generally available more frequently, with the exception of some geographical regions, the most common alternative is to use spatial disaggregation methods combined with EO data (i.e., top-down approach). In this sense, several research institutions have produced gridded population maps at the global scale. Some examples are: the Gridded Population of the World (GPW) offers 5-year interval population estimates at about 1-km^2^ resolution using the areal-weight interpolation [12], the WorldPop provides annual estimates from 2000 to 2020 at approximately 100-m resolution applying statistical methods [26], the Global Human Settlement population grid (GHS-POP) employs the dasymetric method to offer population estimates for four times steps at 250-m resolution relying on built-up areas and GPW (v4.10) population [27], the High Resolution Settlement Layer (HRSL) gives the number of people living within 30-m grid tiles using the dasymetric method [28] and the LandScan estimates annual ambient population over the course of the day since the year 2000 with statistical methods [29]. A recent study reviewed and compared these, and some other large-scale population datasets, highlighting the importance to consider the quality aspects and metadata carefully before selecting a dataset for the target application [30]. However, when it comes to regional or sub-regional applications, the suitability and comparability of these datasets are limited [e.g., 31–34].

Thus, the research landscape provides various contributions on implementing and developing methods for the spatial disaggregation of population at the national, regional and local scales, using simple to cutting-edge techniques with few to many auxiliary data. These *top-down methods* can be generally divided into methods without ancillary information and those relying on ancillary information. The first group consist of rather simple methods where geodata is unavailable. The *areal weighting interpolation* disaggregates population counts equally based on the area of the target zones [35]; and the *pycnophylactic interpolation* iterates the disaggregates in order to generate smoother results while preserving the total population count [36]. Methods from the second group are more up-to-date, since the advance in EO data availability, new methods, and computational power have fostered the production of geospatial auxiliary variables. Within this group, three main methods can be conceptualized, which are explained in more detail in methods section. The *dasymetric method* employs geospatial auxiliary variables to allocate the population, which can be binary if it relies on built-up areas or volume [11, 16, 17, 20, 21, 37], categorical if it considers the land use [20, 38, 39] or it can be based on the built-up density [21, 40]. A recent contribution is *Disaggregator*, a tool that automatizes and facilitates the dasymetric disaggregation of geodata [41]. Then, the *statistical methods* rely on geospatial auxiliary variables to build a *model*—either ordinary least squares, geographic weighted regression or random forest regression—that can be used as weight layer to redistribute the population counts from aggregated source zones to target zones [18, 42, 43]. Finally, *hybrid methods* combine the dasymetric method with weights from the statistical method to refine the population disaggregation, assuming that the weights represent the distribution of the population density within built-up areas. When compared to other methods, the hybrid method seems to produce better results at the expense of greater computational efforts [16, 44, 45].

Accordingly, some authors have conducted comparative analyses of different methods and datasets. For example, Grippa et al. [42] performed ten experiments by performing dasymetric and statistical methods with medium and very high-resolution data. Results were validated with official reference data at one spatial level using two accuracy metrics. Similarly, Reed et al. [16] compared dasymetric, statistical, and hybrid methods using three different globally available urban masks and measured the performance by comparing two accuracy metrics. Besides, Steinnocher et al. [17] compared top-down and bottom-up methods for two cities with different data availability levels and one evaluation metric. On the other hand, Wan et al. [46] examined the performance of alternative sources of ancillary data, including imperviousness, land cover, road networks and night-time lights, on the accuracy of the dasymetric method. Only two studies performed a multi-fold validation, evidencing a high influence in the measured accuracies [11, 21]. However, none of these studies made a complete comparison of all the factors influencing the evaluation of disaggregation methods. In this regard, there is a need for studies comparing disaggregation methods with different degrees of data availability and, at the same time, evaluating the effect of the level of the spatial units used in the validation while comparing the effects on the accuracy metrics. A systematization of these influencing factors is needed to be aware of the capabilities and limitations of population disaggregation methods based on the available input data.

Against this background, the growing availability of methods and auxiliary geodata, together with different levels of population source zones available for different countries, makes it challenging to decide which method is most suitable on the basis of the available data, and how the source zones affect the validation process. Therefore, the aim of this study is conceptualizing and evaluating the capabilities of different population disaggregation methods based on different degrees of data availability (from poor- to rich-data scenarios), as well as analysing the influence of the level of the spatial units on the validation and the employed accuracy statistics in the evaluation. The study aims at systematizing whether the efforts to create reliable and fine-grained population distribution maps based on the available input dataset are a necessity or whether simpler methods provide reasonably accurate results. With it, we outline the results of the experiments conducted as guideline for future population mapping in heterogeneous urban landscapes, and discuss their limitations and next steps.

## Material and methods

In the following we outline our experimental set-up: we first introduce the study area. Secondly, we explain how we divided the input data into population counts, urban masks, and spatial covariates (i.e., geospatial auxiliary variables). Thirdly, we introduce the various methods applied: the binary and categorical dasymetric method, the statistical method, and the combination of both (i.e., hybrid method), and we test combinations of data availability degrees. Lastly, for the validation we evaluate different accuracy statistics with a two-fold validation.

### Study area

The study is framed in the city of Medellin, Colombia. Medellin is a perfect example for this experimental research for three main reasons. First, it is with 2.4 million inhabitants in the city core and 3.95 in the metropolitan area a large test sample consisting of: a wide variability of population and structural densities; a great diversity of urban environments and socioeconomic levels, from planned neighbourhoods to informal settlements with spatially segregated socioeconomic strata; and a great topographic variability, from flat areas to steep slopes [47]. Second, the national statistical office of Colombia (*Departamento Administrativo Nacional de Estadística*, DANE) made recently available microdata of the last Census conducted in 2018, which allows aggregating population counts in several geographic levels and performing a two-fold validation. And third, the city of Medellin provides an open data portal that offers a wide range of geodata, which enables to simulate different degrees of data availability in the experiments.

### Census population data and geographic levels

**Fig 1:**
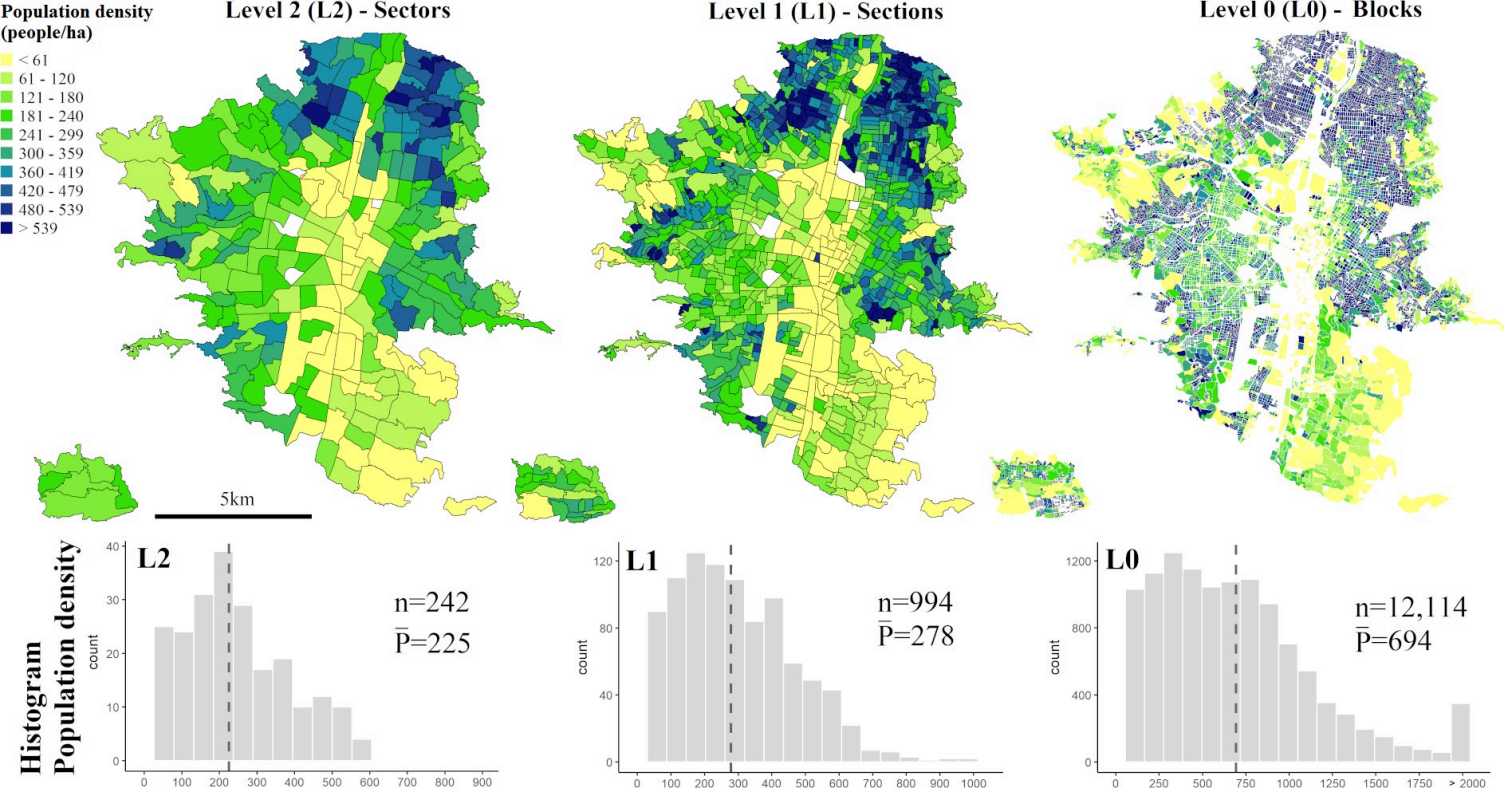
Population density for the three geographic levels (population per hectare). The urban sectors (L2) are used as source zones (SZ), and the urban sections (L1) and census urban blocks (L0) are the validation zones (VZ). At the bottom, the histogram of population density for each level is shown with the number of total spatial units (n) and the mean population density ($\bar{P}$) value with a dashed line.

Microdata from the Colombian National Population and Housing Census 2018 [48] is used to obtain population counts at three geographic levels (i.e., multi-level spatial units). The microdata consist of alphanumeric tables per administrative department with anonymized answers to the Census questionnaires. We use the table “*Personas*” (people) from the Antioquia department. In this table each record accounts for individual members in a household. A combination of fields within the table allows its link to the spatial units provided by the National Geostatistical Framework (*Marco Geoestadístico Nacional*, MGN) [49]. We use three spatial levels with different levels of aggregation: *Urban sectors* (level 2, L2), *urban sections* (level 1, L1) and *census urban blocks* (level 0, L0) (Fig 1). The population data is retrieved by counting the records of the alphanumeric table for each level using a unique identifier. The L2 is used as reference population data (referred as source zones, SZ) and L1 and L0 are used as evaluation data (refereed as validation zones, VZ). As can be seen in Fig 1, population density is predominantly higher in the North and lower in the commercial and industrial corridor in the centre. Mean values of population density vary widely with the level and size of the spatial unit. Reasons for this include the fact that census urban blocks (L0) have less non-built-up areas (e.g., transport infrastructure), increasing built-up density and consequently population density, and due to official institutions dividing denser units into smaller units to keep a more balanced total population per unit. Additionally, we create a regular grid with a spatial resolution of 100 meters (referred from now on as grid) to be used both, as target zones (TZ) where population is estimated and as spatial units in the statistical and combined methods.

### Remotely sensed data

Methods for the disaggregation of population require geospatial auxiliary variables to allocate population. The dasymetric method needs basic information on urbanized areas to know where population can be allocated. Meanwhile, statistical methods require additional spatial covariates to build a model and predict the population distribution to support the disaggregation. Therefore, a series of urban masks and spatial covariates have been issued from remotely sensed imagery and open geodatabases.

#### Urbanized areas

In this study, we use six urban masks with different spatial resolution and dimension to simulate various scenarios of data availability. From medium to high and very-high resolution data, we are able to reproduce a poor-data scenario (using only free satellite imagery), average-data scenario (using a high-resolution satellite imagery and land use information), to a rich-data scenario (where high-detail open geodata exists and optionally three-dimensional information and land use).

The urban masks introduced below are not only used to constrain the allocation of the population to built-up areas, but also to compute spatial covariates used as independent variables in the statistical methods. The covariates are calculated for L2 and for the grid cells (i.e., for the SZ and TZ), pixels or buildings that intersect various spatial units are split and the proportional area is assigned to each spatial unit. The covariates are: *urban density* (den_urb) as the built-up area divided by the area of the spatial unit; *building size density* (den_bs) for rich-data scenarios, where the building size is calculated as the building footprint area multiplied by the number of floors (i.e., total floor area) and then is divided by the area of the spatial unit; *average nearest distance to urban* (dst_urb), which averages the distance in meters from each pixel in the spatial unit to the nearest urban pixel; and a set of spatial metrics measuring area, shape and aggregation properties, such as *mean size* (MS), *shape index* (SI), *porosity* (P), *compactness* (C), *effective mesh size* (EMS), *object densit*y (DO), *dispersion* (DI), *Euclidean nearest neighbour distance* (ENND) and *normalized area-weighted mean standard distance* (AWSDn). The metrics are normalized to diminish the influence of the mapping unit. Definitions and formulas can be found in Sapena [50] (pp. 118–212).

**Fig 2:**
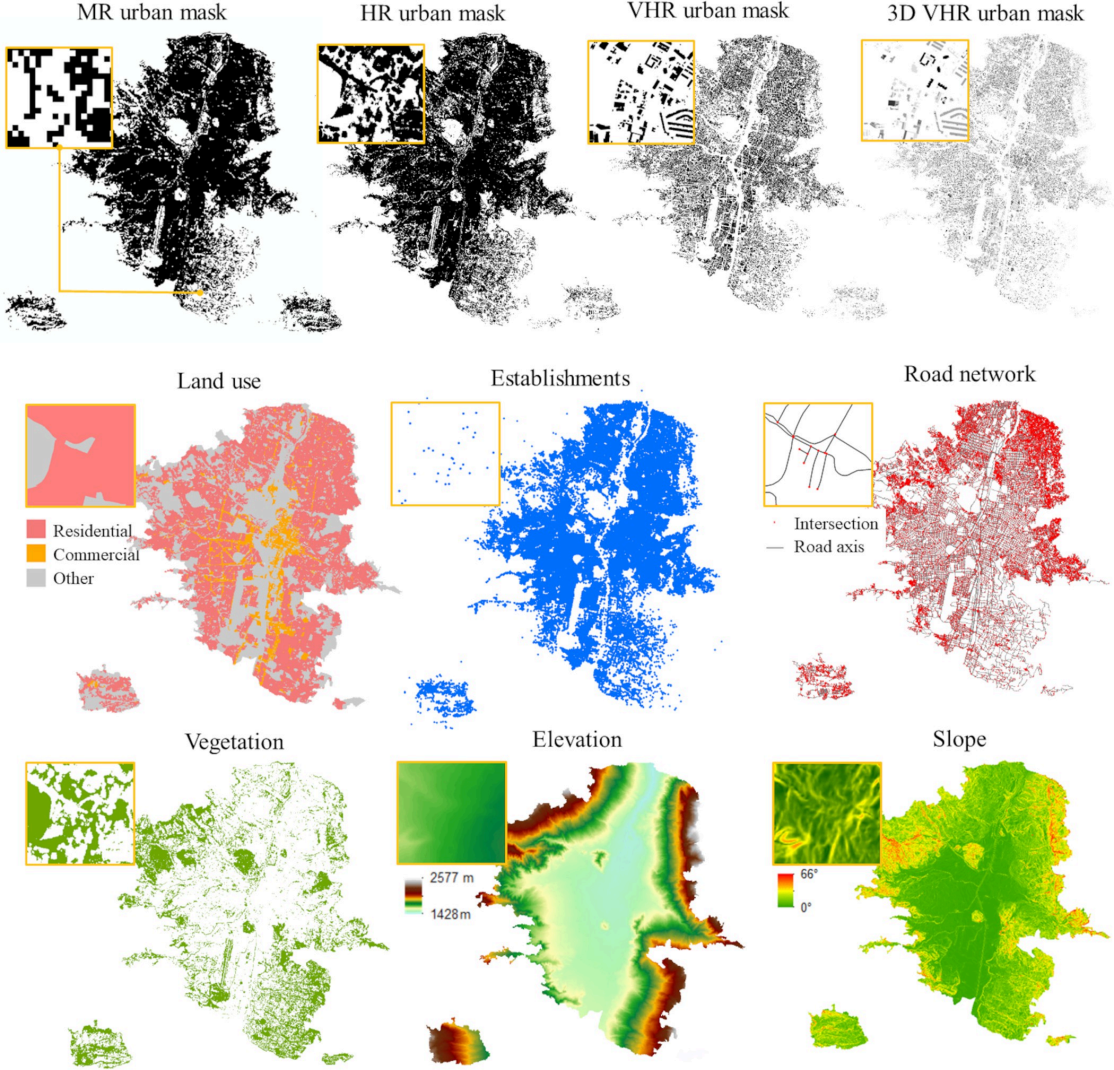
Geospatial covariates. Four urban masks and datasets issued from remotely sensed imagery and open geodatabases. The zoomed areas correspond to the same location (6°11’35.6"N, 75°34’17.7"W).

*Medium-resolution urban mask*. For the poor-data scenarios, we rely on medium resolution (MR), freely available Landsat data. A Landsat-8 image mosaic for the year 2018 is used to produce an urban mask with an approximate pixel size of 30 meters. The image was classified into five land covers (urban, low-vegetation, high-vegetation, bare soil, and water) with an overall accuracy (OA) of 93.61%, as presented in Kühnl et al. [51]. From this product we use the urban land cover with user and producer’s accuracies of 89.97% and 99.79%, respectively (Fig 2).

*High-resolution urban mask*. For the average-data scenarios, we rely on high resolution (HR) Planetscope data, available through a research and educational license [52]. A Planetscope image mosaic for the year 2019 is employed to create an urban mask with a pixel size of 3 meters. A basic pixel-based land cover classification is performed by a random forest algorithm using the variables: red, green, blue and near infrared bands, normalized difference vegetation and water indices (NDVI and NDWI), and elevation data. A training subset of 100,000 points is randomly selected from sampled polygons created by visual photointerpretation to train the model, and a testing subset of 100,000 points is used to assess the accuracy of the classification. Once the model is validated and the accuracy measured, the full set of ground truth samples (more than 350,000) is used to create the final land cover map. The map reached an OA of 96.19%, and the land covers were classified with high user and producer’s accuracies, respectively: urban (96.26%, 97.07%), high-vegetation (98.11%, 98.75%), low-vegetation (94.11%, 94.15%), bare soil (90.70%, 86.51%), and water (97.71%, 94.20%). The urban land cover is extracted and used as urban mask (HR urban mask) (Fig 2).

*Very high-resolution urban masks*. For the rich-data scenarios, we rely on very high resolution (VHR) not always freely available cadastral data. The cadastre database is used to create two urban masks. The data consist of building footprints in shapefile format with attributes such as the number of floors. It was obtained from the City Hall Opendata Service ‘*GeoMedellín*’ in September 2020 [53]. This layer is distinguished from the other masks since it only includes the buildings, while MR and HR urban masks include soil sealing, pavement and road infrastructure. However, cadastre buildings have a handicap in the representation of fast growing and informal developments that are not totally captured [54]. Based on these data, six very high-resolution urban masks are produced. An urban mask with building footprints (VHR), a 3D building mask (3D VHR) using the number of floors to obtain the total floor area (Fig 2), building footprints with land use (VHR LU) and 3D buildings with land use (3D VHR LU).

#### Geospatial covariates

Land use data is a valuable input for estimating population distributions. Since its influence can be included in the statistical methods or endow special treatment for different uses in categorical methods (e.g., residential areas are expected to accommodate more population than commercial areas, and green spaces are not populated). We use the urban and rural homogeneous physical zones datasets from *GeoMedellín*. These consist of two shapefiles that subdivide the city of Medellin into homogeneous areas containing information on usage of the buildings (Fig 2). We aggregate land uses into residential, commercial (also includes services), and others (green areas, amenities, public places, etc.). We also use highly detailed information on the location of commercial, industrial and service establishments. This allows to test whether this kind of high detailed datasets improves results in the disaggregation of population. The dataset was obtained from *GeoMedellín*, and it consists of a point shapefile with georeferenced active establishments (Fig 2).

Based on these data, we calculate covariates for the statistical method for L2 and the grid cells. For the MR and HR analyses, we calculate the *density* and *average nearest distance to residential* (den_res, dst_res), *commercial* (den_com, dst_com) and *other* land uses (den_oth, dst_oth). For the VHR analyses, we assign a land use to each building based on their overlap, and calculate *density* and *average nearest distance to residential buildings (*den_res_b, dst_res_b*)*, *commercial buildings* (den_com_b, dst_com_b) and *other buildings* (den_oth_b, dst_oth_b). Similarly, we derive the *density of building size* for each land use (den_res_bs, den_com_bs, den_oth_bs). The *density* and *average nearest distance to establishment* (den_est, dst_est) are also calculated.

Different configurations of the road network are expected to be observed in areas with different population densities. Therefore, the road network was obtained from *GeoMedellín*, and both, the road axes and their intersection are included as covariates in the model (Fig 2). We calculate the *density* and *average nearest distance* to *road axes* (den_roa, dst_roa) and *intersections* (den_int, dst_int) for L2 and grid cells units.

The amount of green spaces may influence the density of population. Therefore, vegetated areas are included in the analysis. We extract high and low-vegetation areas from the land cover classification (Fig 2). The *density* and *average nearest distance to vegetated areas* (den_veg, dst_veg) are included as covariates.

Finally, Medellin is located within a valley with steep slopes and notable elevation differences. The topography influences the attractiveness of the location (exposure to landslides, centrality, etc.) and thus influences who is settling where. In turn it influences the type of buildings and densities and thus the density of population. Therefore, we use a digital elevation model with 5-meter resolution from *GeoMedellín*, from which we derive a slope map (Fig 2). We calculate the *mean* and *standard deviation* for L2 and grid cells for the *elevation* (m_dem, sd_dem) and *slope* (m_slo, sd_slo). For the computation of geospatial covariates, we used the proportional area of the element covered by the spatial unit of analysis (i.e., L2 and grid cells), with the exception of *elevation* and *slope* covariates where the statistics are calculated for pixels whose centres are within the spatial units.

### Methods for the spatial disaggregation of population

**Fig 3:**
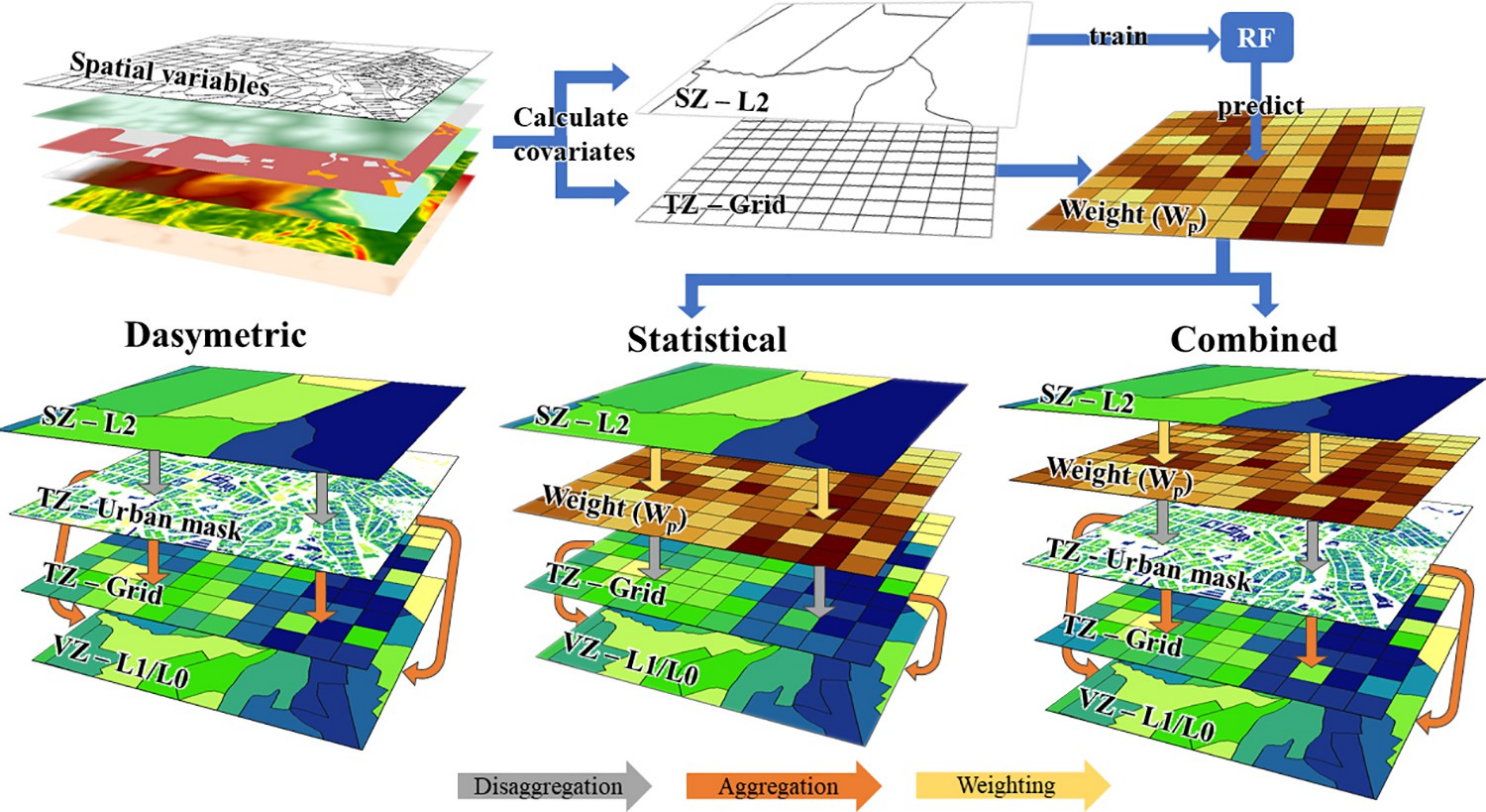
Basic outline of the dasymetric, statistical and combined methods for population disaggregation. Dasymetric: population from source zones (SZ) are distributed to the urban masks at medium, high and very high resolutions (MR, HR, VHR) and to grid target zones (TZ). The result is two-fold validated using the validation zones (VZ) at levels 1 and 0 (L1, L0). Statistical: population from SZ is used as dependent variable to predict the population density at grid level by means of several covariates. The prediction is used as weight (W_p_) to distribute the population into the grid TZ, which is validated using the VZ. Combined: the distribution from SZ to TZ using the weight layer is constrained by the urban masks.

In the following, we describe the different top-down methods tested in this study for downscaling population counts from aggregated source zones (SZ) into single target zones (TZ) of finer spatial resolutions. All the methods presented meet the pycnophylactic property [36], which means that the exact sum of population from the TZ is preserved for each SZ. We use two different TZ, the urban masks themselves and the grid. The latter produces high resolution population maps which are spatially comparable outputs for the different methods (Fig 3). The grid is also used for the statistical method to predict the population density used as weight in the disaggregation.

It is important to note that the population data derived from Census offers population counts based on the place of residence. Therefore, the methods here proposed redistribute the population assuming that everyone is at home, and thus, it refers to night-time population.

#### Binary dasymetric disaggregation

The binary dasymetric disaggregation divides the coarser aggregated population count across built-up areas based on their area or building size. The disaggregation is constrained by the urban mask; therefore, population cannot be attributed to non-urban areas. This method provides a refined population map compared to the source data; however, it assumes the same population density within the L2 spatial unit and depends on the reliability of the urban masks.

**Fig 4:**
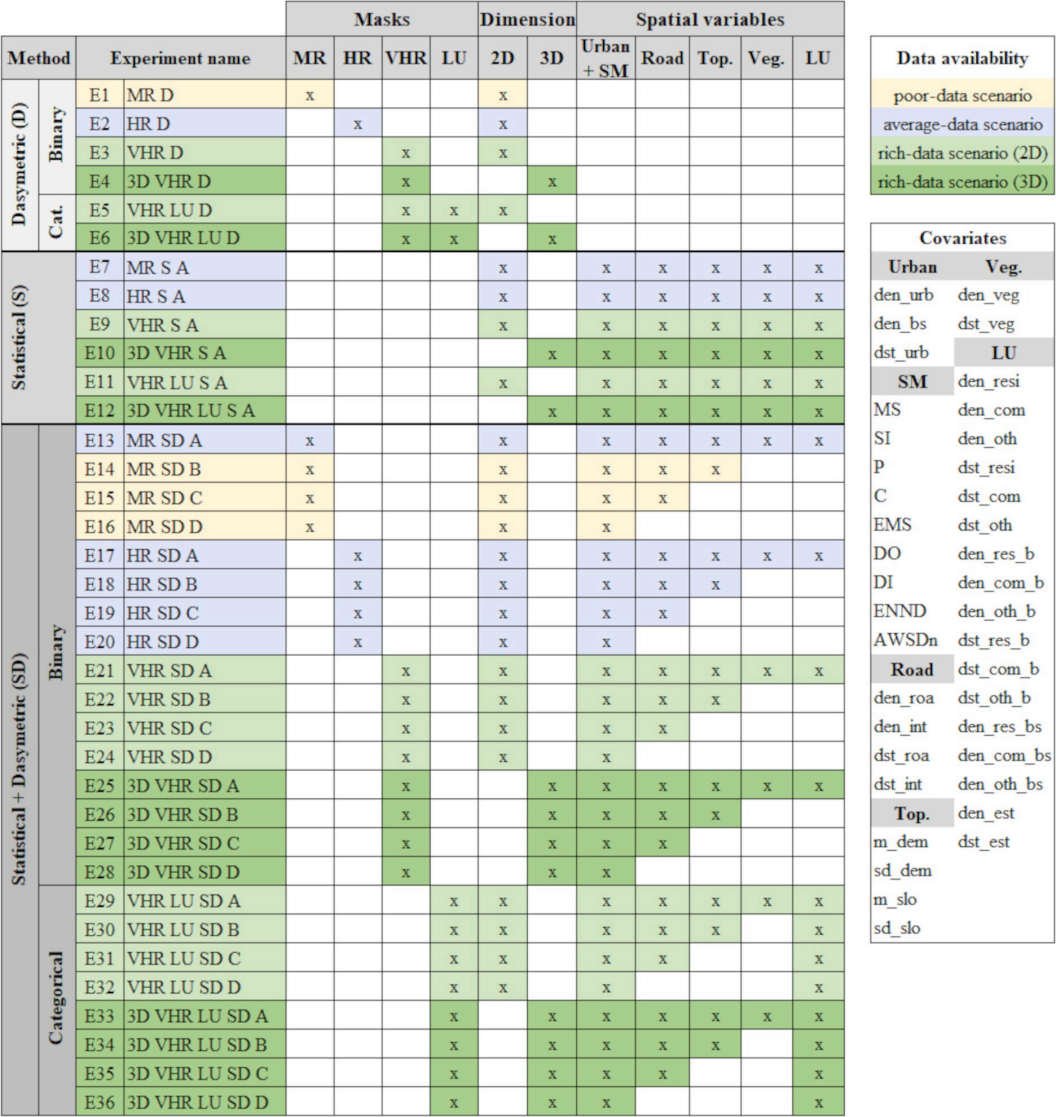
Experiments design. Combination of urban masks, dimensions and covariates to simulate scenarios with poor- (E1, E14-E16), average- (E2, E7-8, E13, E17-E20) and rich-data (E3-E6, E9-E13, E21-E36) for population count disaggregation, by means of binary and categorical dasymetric, statistical and combined methods. The cross (x) indicates the data available for each experiment.

The population from the SZ is equally distributed to the TZs within the particular SZ. The distribution is based on their urban area or building size (Eq 1). Then, the given population at the pixel or building level from the urban mask TZ is aggregated into the grid TZ. This corresponds to the experiments E1-E4 (Fig 4).

$$P_{TZ}=A_{TZ}\cdot \frac{P_{SZ}}{\sum A_{TZ}}$$ (1)

where *P*_*TZ*_ is the calculated population on the TZ (i.e., urban pixel or building), *A*_*TZ*_ is the urban area or building size of the TZ within the SZ, *P*_*SZ*_ is the total population on the SZ.

#### Categorical dasymetric disaggregation

The categorical dasymetric disaggregation follows the same approach as the binary one, but instead of a binary urban mask it relies on a categorical map to apply different rules. In this case, we utilize the land use to impute different occupancy degrees to residential, commercial, and other land uses. Since these types of data are quite detailed and they are still only scarcely available in many regions across the globe, we only implement the categorical approach for the rich-data scenarios (i.e., VHR LU and 3D VHR LU).

The land use of a building only determines its main purpose. In turn mixed usages within buildings cannot be mapped quantitatively. Therefore, residential buildings are considered fully habitable, while commercial and other buildings are partially habitable. Thus, the population from SZ is distributed to the TZ considering the area or building size and their land use by means of weights (Eq 2).

$$P_{TZ}^{LU}={(A}_{TZ}^{LU}\cdot W_{LU})\cdot \frac{P_{SZ}}{\sum_{LU}^{\left(R,C,O\right)}{(A}_{TZ}^{LU}\cdot W_{LU})}$$ (2)

where $P_{TZ}^{LU}$ is the calculated population on the building TZ for a given land use (LU), namely residential (R), commercial (C) and others (O), $A_{TZ}^{LU}$ is the area or building size of a building TZ within the SZ for a given LU, and *W*_*LU*_ is the weight of the LU. $P_{TZ}^{LU}$ (Eq 2) equals *P*_*TZ*_ (Eq 1) when there is only one land use in the SZ.

Regarding the weights, for residential buildings we apply a weight of 1 (*W*_*R*_ = 1, 100% inhabited). However, to obtain the most suitable weights for commercial and other buildings, we combine iteratively different occupancy proportions, from 100% to 0%, and compare the values of the normalized root-mean-square errors (RMSE), the relative total absolute errors (RTAE) measured at L1 and L0, and the over- and underestimation errors at L0. The occupancy rate is selected by minimizing the errors between estimated and reference population using both the area and building size urban masks on both validation levels. We analysed and compared the minimum errors at L1 and L0 and found that occupancy rates between 10% to 40% of commercial and other land uses provide the best results, presenting slight differences in the accuracy metrics between the two levels. Finally, the best combination found was an occupancy of 40% for commercial buildings and 30% for other buildings (*W*_*C*_ = 0.4, *W*_*O*_ = 0.3), the results of the analysis can be found in the S1 and S2 Figs. This method corresponds to the experiments E5-E6 (Fig 4).

#### Statistical disaggregation

In the statistical method we use predictive models to estimate population density distribution at a finer spatial scale. The predicted map is used as a weight to allocate population. This avoids the equal population density within SZs obtained in dasymetric methods. Ideally, this weight layer reflects the underlying mechanisms of population distribution better [18]. In order to obtain the weight, we use the random forest regression algorithm [55] with 500 trees, 3 variables at each split and 5 terminal nodes, using the *randomForest* package in R [56], since it performed better than the multiple linear regressions. We calculate the population density at SZ and used this as independent variable. In our case it provides better accuracies than using the natural log, unlike other studies [e.g., 18, 42]. The model is trained at SZ level. We trained with different subsets of covariates based on the experiments and data availability degrees (Fig 4). To improve the performance of the models, we discarded highly correlated covariates by selecting the one with higher correlation with the dependent variable and covariates correlated with the size of the SZs. The internal accuracy metrics of the random forest models are reported for each experiment E7-E36. Then, the trained model is used to predict the population density at grid TZ level (Fig 3, middle). The result is the weight (*W*_*P*_) used to disaggregate population from the SZs to TZs (Eq 3).

$$P_{TZ}^{P}=W_{P}\cdot \frac{P_{SZ}}{\sum W_{P}}$$ (3)

where $P_{TZ}^{P}$ is the calculated population on the grid TZ based on the predicted map (P), and *W*_*P*_ is the weight of population density for the TZ based on the predicted population density.

#### The combined statistical and dasymetric disaggregation

We combine the statistical method with the binary and categorical dasymetric disaggregation by including *W*_*P*_ in the formulas and constraining the allocation of the population to the different masks (Fig 3, right), as shown in Eq 4 for a binary approach and Eq 5 for a categorical approach:

$$P_{TZ}^{Pc}={(A}_{TZ}\cdot W_{P})\cdot \frac{P_{SZ}}{\sum {(A}_{TZ}\cdot W_{P})}$$ (4)

$$P_{TZ}^{LU-Pc}={(A}_{TZ}^{LU}\cdot W_{LU}\cdot W_{P})\cdot \frac{P_{SZ}}{\sum_{LU}^{\left(R,C,O\right)}{(A}_{TZ}^{LU}\cdot W_{LU}\cdot W_{P})}$$ (5)

where $P_{TZ}^{Pc}$ and $P_{TZ}^{LU-Pc}$ are the calculated population on the TZ (i.e., urban pixel or building) based on the predicted map and constrained by the urban masks (Pc) using the binary and categorical (LU) approaches, respectively.

### Validation approach

As described above, census population counts at L2 are used as reference data, while counts at L1 and L0 are used as evaluation data. For the assessment of the efficiency of the different disaggregation methods and the data availability degrees, we calculate the accuracy metrics by confronting the reference population at VZ to the estimated population at VZ. The estimated population at VZ is measured by aggregating the estimated population on TZ (pixels or buildings) for each VZ spatial unit. It is important to note that we do not have georeferenced point population data and thus we cannot explicitly validate the results. Instead, we assess the efficiency of the reallocation of the population [42]; however, the validation at L0 approximates a bottom-up validation since it provides the population in each urban block, which is the smallest administrative unit (covering only a few buildings, green spaces and pavement).

For the quantitative assessment of accuracies, we use different accuracy metrics to compare their performance and suitability (Table 1). The *coefficient of determination (R*^*2*^*)* quantifies the explained proportion of the population variance (Eq 6), the *normalized root-mean-square error (RMSE)* uses the mean population at VZ for normalization of the standard deviation of residuals (Eq 7), the *relative total absolute error (RTAE)* is the ratio between the sum of all absolute errors and the total population (Eq 8), and *the mean absolute percentage error (MAPE)* measures how accurate a forecast is by comparing the error against the population at the validation unit (Eq 9). Additionally, since the VZ L0 consists of spatially non-continuous urban blocks with unpopulated areas in between (Fig 1), contrary to L2 and L1 that are continuous, we can quantify the *overall underestimation (UE*, Eq 10*)* and *overestimation errors (OE*, Eq 11*)* between SZ L2 and VZ L0. The former occurs when census urban blocks have official population counts but there are neither urban pixels nor buildings and thus the estimated population is zero. The latter occurs when population is estimated outside urban blocks because there are urban pixels or buildings while the official population count is zero.

**Table 1:** Relative accuracy statistics for the validation of the population disaggregation methods.

Accuracy statistic	Equation	
**Coefficient of determination (R** ^**2**^ **)**	$R^{2}=1-\frac{{\sum_{i=1}^{n}\left(P_{{VZ}_{i}}-P_{{VZ}_{i}}^{e}\right)}^{2}}{{\sum_{i=1}^{n}\left(P_{{VZ}_{i}}-\bar{P}\right)}^{2}}\cdot 100\;(\%)$	(6)
**Normalized root-mean-square error (RMSE)**	$RMSE=\frac{1}{\bar{P}}\cdot \sqrt{\frac{{\sum_{i=1}^{n}\left(P_{{VZ}_{i}}-P_{{VZ}_{i}}^{e}\right)}^{2}}{n}}\cdot 100\;(\%)$	(7)
**Relative total absolute error (RTAE)**	$RTAE=\frac{1}{P}\cdot \sum_{i=1}^{n}\left|P_{{VZ}_{i}}-P_{{VZ}_{i}}^{e}\right|\cdot 100\;(\%)$	(8)
**Mean absolute percentage error (MAPE)**	$MAPE=\frac{1}{n}\cdot \sum_{i=1}^{n}\left|\frac{P_{{VZ}_{i}}-P_{{VZ}_{i}}^{e}}{P_{{VZ}_{i}}}\right|\cdot 100\;(\%)$	(9)
**Underestimation error (UE)**	$UE=\frac{1}{P}\cdot \sum_{{{(P}_{VZ}^{e}=0)}_{i}}^{n}P_{{VZ}_{i}}\cdot 100\;(\%)$	(10)
**Overestimation error (OE)**	$OE=\frac{1}{P}\cdot (P-\sum_{i=1}^{n}P_{{VZ}_{i}}^{e})\cdot 100\;(\%)$	(11)
**Absolute percentage error (APE)**	${APE}_{i}=\left|\frac{P_{{VZ}_{i}}-P_{{VZ}_{i}}^{e}}{P_{{VZ}_{i}}}\right|\cdot 100\;(\%)$	(12)

Where *P*_*VZ*_ is known population at VZ (L1 or L0), $P_{VZ}^{e}$ is the estimated population at VZ, *n* is the number of VZ spatial units, *i* is a given VZ spatial unit, $\bar{P}$ is the mean known population at VZ and P is the total known population.

Finally, in order to understand better the potential causes of errors in the disaggregation, we analyse *the absolute percentage error* (*APE*, Eq 12) in relation to the area, population density, and primary land use of the VZ L0 spatial units. We do so by grouping the errors into quartiles based on the area, population density and land use shares to identify differences in the errors. This is conducted by means of the Wilcoxon rank-sum test, since the errors do not follow a normal distribution. We ascertain whether the distribution of errors is significantly different between groups and, therefore, we can reject the null hypothesis that the distributions are equal [57].

### Experiments design

Fig 4 summarizes the systematic experiments conducted in this study by combining availability data degrees (poor, average and rich) and methods (from basic to complex) used to perform thirty-six different scenarios. For each experiment (E), the resolution of the urban mask is indicated (MR, HR and VHR), as well as additional attributes such as three-dimensional information (3D) or land use (LU). Besides, the geospatial auxiliary information is grouped into spatial metrics (SM), road network (Road), topography (Top.), vegetated areas (Veg.) and LU. The right table shows the covariates created from the auxiliary information which are included in the statistical and combined methods. For the statistical and combined methods, we conduct four experiments for each mask by increasing the number of available covariates (A, B, C and D, from more to less data). For the statistical method, only the case A is reported to shorten the experiment list, since the results were not promising.

### Best population grid map

As a key result, the best population grid map was created using the method of all the experiments that provided the best outcome and the L0 as SZ. In order to assess the usability in relation to the other results, we compared the best map with the population grid maps of each experiment at the various resolution levels. From this, we want to derive statements about which data and methods can be expected to produce which results and whether additional effort in these domains is worthwhile for population estimation.

## Results

In this section, the experimental results are presented structured by the resolution of the input data and the different availability levels of auxiliary geodata. Subsequently, the errors are analysed, and lastly, the final grid population maps are compared against the best population grid map. Results with the estimated population for all the experiments and the best population grid map that support the findings of this study are available on Figshare: https://doi.org/10.6084/m9.figshare.c.5857320.v1.

### Population disaggregation at medium resolution

The experiments at medium resolution are based on the Landsat-8 urban mask and a subset of covariates conceptualized as poor-data availability and as average-data availability when land use data are included.

The first experiment (E1, Table 2) corresponds to the simple disaggregation approach where population is equally allocated to urban pixels. In this case, the validation at level 1 (VZ L1, Table 2) shows a reasonable high R^2^, an RMSE lower than 30%, while the RTAE shows with regards to the total population a total error of 20%. This indicates an intermediate error, which may suggest a good performance in the disaggregation; however, the MAPE that measures the percentage error relative to the population in VZ shows the opposite. The validation at level 0 (VZ L0, Table 2) shows a worse performance in comparison to L1, since the R^2^ is lower than 50%, the RMSE and RTAE are reporting considerable high errors, and there is a huge population overestimation (OE). This means that almost 34% of the population was allocated in urban, but non-populated, areas according to the official census. The MAPE shows a similar behaviour in both validation levels.

**Table 2:** Accuracy metrics for the population disaggregation at medium resolution.

		RF–SZ L2		VZ L1				VZ L0					
Experiment		R^2^	RMSE	R^2^	RMSE	RTAE	MAPE	R^2^	RMSE	RTAE	MAPE	OE	UE
**E1**	**MR D**	-	-	83.26	28.85	20.16	82.85	45.50	85.99	50.11	81.20	33.85	0.37
**E7**	**MR S A**	84.52	26.27	85.29	27.04	18.06	60.74	29.99	97.46	52.36	84.17	30.82	0.00
**E13**	**MR SD A**	84.52	26.27	87.08	25.34	17.13	65.42	51.82	80.85	46.76	69.10	33.17	0.37
**E14**	**MR SD B**	78.65	30.85	84.67	27.60	18.75	76.46	46.81	84.95	48.86	74.57	34.09	0.37
**E15**	**MR SD C**	78.65	30.85	81.97	29.94	20.11	78.25	42.92	88.00	49.56	74.26	34.38	0.37
**E16**	**MR SD D**	22.06	58.52	82.94	29.12	20.26	83.27	44.91	86.45	49.93	80.60	33.77	0.37

Results of the random forest regression models trained at the source zones level (RF–SZ) and accuracy metrics for the validation of the population disaggregation at level 1 (VZ L1) and level 0 (VZ L0) for medium resolution data corresponding to the poor- and average-data availability scenarios (values are in percentages).

The second experiment (E7, Table 2) relied on the statistical method, where the random forest regression model reached a high accuracy using all non-correlated covariates (R^2^ = 84.5%). The most important covariates in the model were the road network, topography, vegetation and land use related, while the spatial metrics and the distance to urban areas are less relevant. This is the reason why discarding land use, topography and road network covariates provides a worse model (E16). The disaggregation at L1 shows an improvement against the dasymetric method (E1), providing the lowest MAPE of all experiments in the medium resolution scenarios (MAPE = 60.7%) due to less population overestimation within L1 units. However, at L0, the population disaggregation shows a worse result, even if the OE and UE are the lowest in this resolution level, the other accuracy metrics show a poor performance (Table 2).

With regards to the combination of statistical and dasymetric methods (E13-16), we reached the best result in case A, when all covariates are used at both levels, L1 and L0. Besides, only cases A and B (E13 and E14) have a better result than the E1, which means that the covariates from the urban mask and the road network alone do not improve the disaggregation, but their usage improves the performance in cases A and B.

### Population disaggregation at high resolution

The experiments at high resolution, which are based on the Planetscope urban mask and a subset of covariates, belong to the average-data availability scenarios. Within this group, the dasymetric disaggregation (E2) not only provides similar results to E1 (MR) both at L1 and L0, but also R^2^ and RMSE reveal a slightly worse outcome (Tables 2 and 3). This somehow surprising result can be related to the urban mask, since the finer-grain pixels introduce more noise and non-populated urban pixels such as transport infrastructure and pavement. These classes are also the reason for the high OE values. Concerning the statistical and combined experiments, the random forest algorithm performed well when almost all covariates were used (cases A and B); on the contrary, the spatial metrics and the urban mask themselves cannot predict population density (case D, R^2^ = 7.8%). Like in the MR experiment, the MAPE in the statistical method (E8) at L1 shows the lowest relative error, but the validation at L0 indicates a misperception. The accuracy metrics of the statistical method at L1 are considerably better than the dasymetric one, but this trend does not uphold at L0, which may wrongly imply a good population disaggregation result when using only validation data at L1 or contiguous spatial units. Regarding the results of the statistical and dasymetric method we achieve the best population maps (E17 and E18) at both levels, also improving the performance of the best population map in the MR experiments and diminishing the errors (Table 3).

**Table 3:** Accuracy metrics for high resolution population.

		RF—SZ L2		VZ L1				VZ L0					
Experiment		R^2^	RMSE	R^2^	RMSE	RTAE	MAPE	R^2^	RMSE	RTAE	MAPE	OE	UE
**E2**	**HR D**	-	-	82.57	29.44	20.04	86.85	45.06	86.34	49.35	79.57	33.29	0.01
**E8**	**HR S A**	83.22	27.69	85.64	26.72	17.89	59.00	30.81	96.89	52.21	83.68	30.79	0.00
**E17**	**HR SD A**	83.22	27.69	89.07	23.31	15.60	63.48	55.15	78.01	44.84	65.43	32.31	0.01
**E18**	**HR SD B**	78.71	31.00	85.30	27.04	17.90	75.56	47.90	84.08	47.37	70.94	33.44	0.01
**E19**	**HR SD C**	61.70	40.42	82.47	29.52	19.56	77.59	43.50	87.55	48.20	70.77	33.80	0.01
**E20**	**HR SD D**	7.87	63.35	82.88	29.17	20.12	84.43	45.39	86.07	49.29	78.82	33.33	0.01

Results of the random forest regression models trained at the source zones level (RF–SZ) and accuracy metrics for the validation of the population disaggregation at level 1 (VZ L1) and level 0 (VZL0) for high resolution data corresponding to the average-data availability scenarios (values are in percentages).

### Population disaggregation at very high resolution

In the experiments at very high resolution we produced several population maps. The first group employs binary data on building area and building size (i.e., total floor area) as urban masks and covariates (Table 4). The second group includes information on the main land use of buildings (Table 5) being part of the rich-data availability scenarios.

**Table 4:** Accuracy metrics for the population disaggregation at very high resolution.

		RF—SZ L2		VZ L1				VZ L0					
Experiment		R^2^	RMSE	R^2^	RMSE	RTAE	MAPE	R^2^	RMSE	RTAE	MAPE	OE	UE
**E3**	**VHR D**	-	-	84.38	27.86	18.05	74.70	53.62	79.32	36.42	73.17	8.00	0.21
**E9**	**VHR S A**	88.62	22.58	83.58	28.57	18.44	60.14	26.94	99.56	52.51	83.66	30.58	0.00
**E21**	**VHR SD A**	88.62	22.58	79.55	31.89	18.56	59.05	51.59	81.04	38.42	65.77	6.68	0.21
**E22**	**VHR SD B**	86.75	24.69	76.42	34.24	19.76	65.77	45.33	86.13	40.10	69.01	6.88	0.21
**E23**	**VHR SD C**	86.54	24.49	74.51	35.60	20.37	65.93	42.67	88.20	41.01	69.45	6.82	0.21
**E24**	**VHR SD D**	86.67	24.43	73.25	36.47	20.92	65.53	41.77	88.88	41.68	69.87	6.67	0.21
**E4**	**3D VHR D**	-	-	90.28	21.98	14.90	57.64	65.55	68.36	30.86	59.44	6.46	0.21
**E10**	**3D VHR S A**	89.03	22.48	83.88	28.31	18.31	59.41	27.15	99.42	52.43	83.41	30.53	0.00
**E25**	**3D VHR SD A**	89.03	22.48	90.69	21.52	14.35	43.44	72.18	61.43	31.84	54.81	5.44	0.21
**E26**	**3D VHR SD B**	87.21	23.90	88.90	23.50	15.42	49.66	66.65	67.27	33.54	57.61	5.64	0.21
**E27**	**3D VHR SD C**	86.96	24.36	87.61	24.82	16.12	50.67	65.03	68.88	34.47	58.17	5.59	0.21
**E28**	**3D VHR SD D**	87.07	24.09	87.20	25.23	16.54	50.16	64.57	69.33	35.01	58.60	5.48	0.21

Results of the random forest regression models trained at the source zones level (RF–SZ) and accuracy metrics for the validation of the population disaggregation at level 1 (VZ L1) and level 0 (VZL0) for very high-resolution data (2D and 3D) corresponding to the rich-data availability scenarios (values are in percentages).

**Table 5:** Accuracy metrics for the population disaggregation at very high resolution with land use information.

		RF—SZ L2		VZ L1				VZ L0					
Experiment		R^2^	RMSE	R^2^	RMSE	RTAE	MAPE	R^2^	RMSE	RTAE	MAPE	OE	UE
**E5**	**VHR LU D**	-	-	87.37	25.06	15.67	58.77	62.90	70.94	33.44	60.22	6.34	0.21
**E11**	**VHR LU S A**	85.03	26.42	85.50	26.85	17.67	58.94	29.67	97.68	52.03	83.02	30.75	0.00
**E29**	**VHR LU SD A**	85.03	26.42	81.78	30.09	17.37	48.44	56.20	77.09	36.57	58.65	5.67	0.21
**E30**	**VHR LU SD B**	83.86	27.13	81.94	29.96	17.22	47.73	56.48	76.84	36.37	58.56	5.67	0.21
**E31**	**VHR LU SD C**	83.76	26.62	80.81	30.88	17.67	47.27	54.86	78.26	37.20	58.94	5.60	0.21
**E32**	**VHR LU SD D**	83.50	27.06	80.74	30.94	17.91	47.85	54.93	78.19	37.33	59.05	5.58	0.21
**E6**	**3D VHR LU D**	-	-	92.75	18.99	12.57	43.14	74.66	58.63	27.69	48.93	5.15	0.21
**E12**	**3D VHR LU S A**	85.15	26.03	86.23	26.17	17.38	58.42	30.37	97.19	51.86	82.77	30.74	0.00
**E33**	**3D VHR LU SD A**	85.15	26.03	91.74	20.27	13.38	34.83	74.10	59.28	29.97	48.85	4.69	0.21
**E34**	**3D VHR LU SD B**	84.26	26.77	91.75	20.25	13.25	34.23	74.02	59.36	29.88	48.81	4.68	0.21
**E35**	**3D VHR LU SD C**	84.23	27.14	90.98	21.18	13.88	34.51	72.97	60.56	30.67	49.35	4.64	0.21
**E36**	**3D VHR LU SD D**	82.83	27.95	91.15	20.97	13.85	34.36	73.29	60.20	30.49	49.04	4.62	0.21

Results of the random forest regression models trained at the source zones level (RF–SZ) and accuracy metrics for the validation of the population disaggregation at level 1 (VZ L1) and level 0 (VZL0) for very high-resolution data with land use information for the categorical disaggregation (2D and 3D) corresponding to the rich-data availability scenarios (values are in percentages).

Regarding the binary dasymetric methods (E3 and E4, Table 4), the VHR results are considerably better than the MR and the HR results. Although at L1 the improvement may seem rather low, the validation a L0 confirms a considerably better performance. The inclusion of 3D information (E4) considerably improves the population disaggregation, reaching a R^2^ of 65.5% at L0 and a much lower MAPE in comparison to the other experiments. For the statistical methods, we found that at the VHR level the RF regression performs much better. This is even measured when only the spatial metrics and the urban masks (R^2^>86%) are used; however, this does not provide better results in the statistical (E9 and E10, Table 4), nor in the combined methods (E21-E28, Table 4) at both validation levels. In fact, the combined methods are measured with higher errors than the dasymetric ones. The exception is when the RF weight with all covariates (case A) and the building sizes for the dasymetric disaggregation (E25) are used. The improvement compared to the dasymetric (E4) approach is almost unnoticeable for L1, but substantially better for L0. In general, this behaviour suggests that in spite of the good RF model accuracies, the weight layers at the VHR scenarios are not able to improve or refine the distribution of the population at the building level.

The best population maps from all experiments are measured for the VHR categorical dasymetric methods (E5 and E6). The validation results at L1 (R^2^ = 87.4% and 92.8%) and L0 (R^2^ = 62.9% and 74.7%) as well as the lowest measured overestimation of population in unpopulated places (below 6%) reveal its capabilities. Concerning the RF models, the performances are fairly good; although, none of the statistical nor combined methods beat the dasymetric population maps, even if in E33-36 the accuracies are very similar. The only accuracy metric that improves is the MAPE, which could be related to less populated urban blocks having higher errors in the dasymetric method, and thus, increasing the APE and extreme outliers.

### Analysis of errors

For a deeper understanding of errors on population disaggregation methods, we reflect on the APE distribution in relation to the land usage, the size of the spatial unit, and population density. In general, the APEs follow skewed left distributions. This first of all means that the means and medians are not zero centred, and second, there are extreme negative outliers mostly with high overestimations (APE < -100%). We found these anomalous errors in large, dense built-up units of low population densities, i.e., areas that relate to a low presence of residential land use.

**Fig 5:**
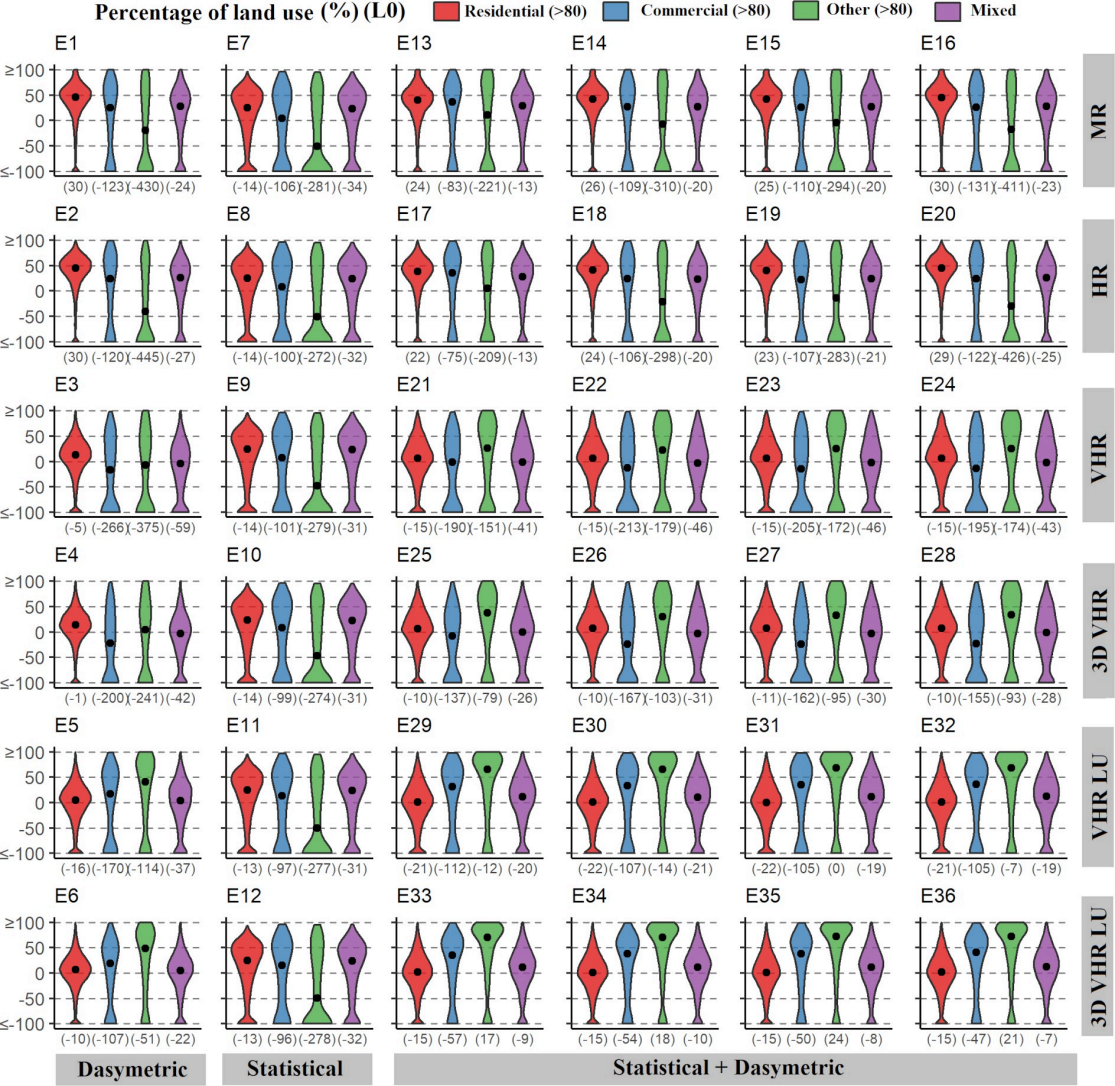
Violin plots with the distribution and density of absolute percentage errors (APE). Errors are grouped by land use percentages by experiment, for the validation L0 using the urban blocks. In red, errors for mostly residential blocks (>80% of the land use is residential, the rest can be commercial and/or others, n = 9,236). In blue and green errors for mostly commercial (n = 307) and other (n = 494) land uses urban blocks, respectively. And purple gathers errors for urban blocks without a predominant land use (n = 2,050). The dot reports the median APE per land use group, while the number between brackets in the x-axis reports its mean.

Fig 5 displays the variation of the errors based on the dominant land use of the urban blocks. We created four groups representing mostly residential (n = 9,236), commercial (n = 307) and other (n = 494) land uses, which represents more than 80% of the areal land use, and urban blocks without a predominant land use (n = 2,050). We observed that errors clearly differ between land uses. Besides, the Wilcoxon rank-sum test confirmed that the distributions of the errors are significantly different within groups. In MR and HR population maps (first and second row, Fig 5), we found slightly higher errors at urban blocks with mainly residential use. There, few outliers were identified and most of the extreme errors are in the commercial or urban blocks of other land uses. This trend changes in VHR population maps, especially with the categorical dasymetric methods (VHR LU and 3D VHR LU, Fig 5). In this case the errors of commercial and other land uses urban blocks are higher than residential and mixed land uses; however, there are fewer extreme errors. The use of weights in the categorical methods considerably improves the estimation in residential and mixed urban blocks, but overestimates population in commercial and other land use urban blocks as reported by the median APE. However, the mean APE and the distribution of errors point fewer outliers and thus better estimates in populated commercial and other land use built-up areas when using weights in the population disaggregation (VHR vs VHR LU, and 3D VHR vs 3D VHR LU, Table 5). Hence, the land use diversity affects population disaggregation methods.

Additionally, we compared the errors based on the area of the spatial unit of validation L0. Higher errors were found at the smaller urban blocks (Q1, S3 Fig), while larger units tend to have lower errors. However, extreme errors are most common in the fourth quartile (Q4, S3 Fig). The errors are significantly different within area-based groups (S3 Fig). Similarly, errors also vary with the population density, especially in MR and HR population maps. The effect is diminished in VHR population, but still the difference between groups are significantly different. Errors tend to be higher in more densely populated urban blocks (Q4, S4 Fig); however, all anomalous errors, i.e., very high overestimations, are concentrated in the least dense units (Q1, S4 Fig). Therefore, not only the land use, but also the area and the population density of the spatial unit influence the accuracy in population disaggregation methods. Thus, fine-grained population maps from heterogeneous urban structures might have higher uncertainties than from homogeneous ones.

**Fig 6:**
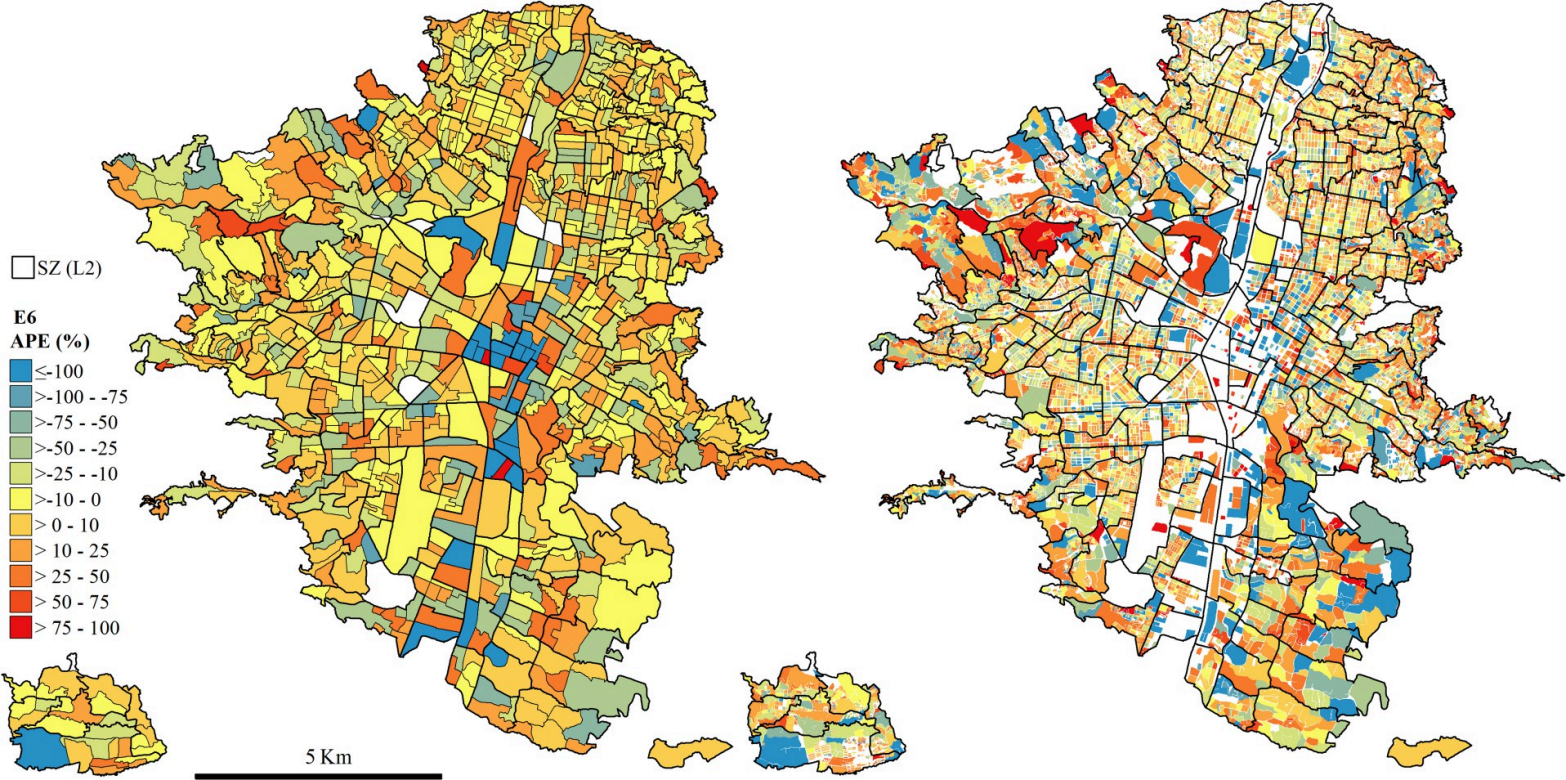
Spatial distribution of absolute percentage errors (APE) for L1 (left) and L0 (right) for the best performing experiment (E6). Yellowish colours indicate low errors in population disaggregation, while dark blue and red show high underestimation and overestimation of population, respectively. The boundaries of the L2 source zones (SZ) used in the disaggregation are shown in bold on the top of the two maps.

We specify the errors of our best population disaggregation model at L1 and L0 (E6) in Fig 6. We map the spatial distribution of APEs to illustrate the spatial variability of accuracy. At L1, high errors are concentrated around the business and industrial sectors in the Center-South corridor of the city (dark red and blue) that correspond to areas with commercial, service and other land uses (see Fig 2). These areas are characterized by low population densities according to the census (Fig 1). Our model estimated much higher populations in these areas, even if the land use of the buildings is considered. At L0, the errors in these areas are accounted as overestimations (OE) since there is officially no attributed population at many of these urban blocks. At this level, the highest errors are located on the South-East of the city (known as *El Poblado*) and the Western parts. Both correspond to the wealthiest and expansion areas of the city, with high-rise development composed mostly of large units, with a relatively low population density according to the census.

Finally, we created the best population grid map we can achieve with our data by using the census population counts at L0 as source zones, and the categorical dasymetric method with 3D information and land use data (S5 Fig), which corresponds to the method and data from the best performing method (E6). The highly dense areas of the city are located in the North, East and the West edges (dark blue). While the least populated areas (in yellow) are in the business and industrial sectors in the Center-South corridor and in the expansion areas of the city (*El Poblado* on the South-West and *La Loma* and *Pedregal Alto* on the West). Based on this best result we cross-compare it with our L2 SZ grid population maps (E1-36).

**Fig 7:**
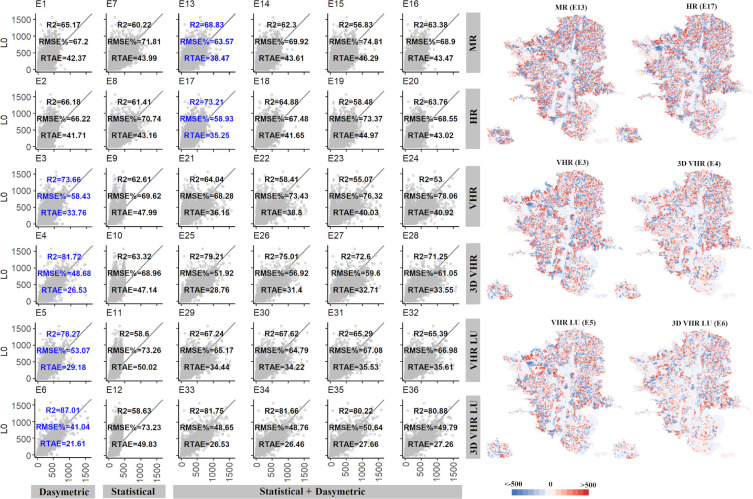
Scatterplots comparing the results of the best population grid (P_L0_, SZ L0, y-axis) against the estimated population map from all experiments (P_e_, SZ L2, x-axis) with the accuracy metrics. On the right, the maps show the spatial distribution of residuals (P_L0_ –P_e_) for the best P_e_ grid by availability level scenario (highlighted in blue). Bright blue colours indicate an underestimation of population per cell, while red show overestimation. Light colours indicate low errors.

Fig 7 shows the usability of population grid maps in relation to the best product. The scatterplots compare the estimated population at grid level for each experiment (*P*_*e*_, x-axis) versus the population derived from L0 (*P*_*LO*_, y-axis). The reported accuracy metrics quantify the errors at the grid level evidencing the performance of each experiment. The right side of Fig 7 shows the maps with the residuals at grid level (*P*_*LO*_-*P*_*e*_) for the best population map of each group of experiments at the various resolution levels (highlighted in blue).

Overall, the spatial distribution of the population is well represented by the results of most of the experiments carried out; however, some limitations are found especially for the MR and HR scenarios. But still, the best result of the MR experiments (E13) has a coefficient of determination of nearly 70% with the best population map and a RTAE of 38.5%. This indicates a high correlation with some restrictions that are illustrated in the map with the distribution of errors (Fig 7, first row right column). The high populated cells are underestimated (dark red) and the overestimation due to the presence of urban pixels in sparsely populated areas is large. Similarly, the HR scenario presents an improvement against the MR scenario, but still the underestimations are quite notable (E17) (Fig 7, second row right column). As previously seen, the improvement of VHR against HR was not very notable. This is verified by the comparison of population grids with very similar accuracies, despite an improvement in RTAE (E3). We found the result of the VHR population disaggregation using the building heights (E4) provides betters results to using the area and the land use (E5). Therefore, the heights of buildings are a better population proxy than their land use. Lastly, the best result of all the experiments using L2 data (E6) shows a very high correlation with the best population map (R^2^ = 87%) and diminishes the errors up to 22% of the total population in Medellin.

## Discussion

There are various methodological approaches to disaggregate aggregated population figures at a coarser scale to higher resolved spatial units. Which approach promises which accuracies, however, has not been systematically investigated for the complex urban space. In this study, we performed an exhaustive experimental research on population disaggregation methods in an heterogeneous urban landscape. We applied five different top-down methods (i.e., binary and categorical dasymetric, statistical, and binary and categorical combined statistical and dasymetric) on different subsets of data with diverse resolutions and degrees of availability. And all the resulting population maps were systematically validated with a two-fold approach using six accuracy metrics.

In general, of course, it is, on the one hand, important to note that the reliability of the top-down population disaggregation methods depend on the confidence of the input population data; therefore, the derived fine-grained population maps can only be as good as the data they rely on [2]. In this case, the census data was a source of controversy due to their differences with the projection of the population [58]; however, the final released product followed a series of revisions and adjustments [59]. On the other hand, the use of remotely sensed datasets and open geodata is also subject to introduce errors in the disaggregation; however, these can be indirectly quantified in the validation process.

Based on the experiments and observations made, we developed a guideline with general tips, but especially for heterogeneous urban landscapes, for attempts to create fine-grained population maps based on the input data:

- As expected, the spatial resolution and three-dimensional information used in population disaggregation methods is decisive in the accuracy of the fine-grained population map. The accuracy substantially increases with the spatial resolution and the detail of the input data. This is in line with the findings of Steinnocher et al. [17] and Grippa et al. [42]. In this study, this is especially noticed in the overestimation of population. Urban masks derived from remotely sensed data resulted in nearly 28% higher population overestimations. This is related to the definition of urban pixels. They include additional information to built-up areas, such as roads, pavements and other infrastructures, and thus attributing people in non-inhabited areas. These errors could be partly reduced by distinguishing building footprints from other infrastructure in the classification of the urban masks [60] or applying postprocessing methods to remove undesired urban pixels. But, in doing so, advantages and disadvantages must also be balanced: in the case of Medellin, remote sensing brings additional useful information on built-up areas in informally urbanized neighbourhoods lacking in the cadastre database.
- The dasymetric method provides the highest accuracies when using highly resolved geodata. That is, using building footprints, the number of stories and land use data. There is one exception when the dasymetric method is combined with the statistical weight approach and the land use is included as covariate. In this specific case, the accuracy metrics are better on the expense of the RTAE, where the dasymetric approach has a lower absolute error with regards to the total population. Therefore, when VHR data are available, the dasymetric method is found to be the best option: the accuracies are better, it takes less computational effort, and it is more straightforward and easier to apply. When land use data are available, the categorical dasymetric approach is found to provide the best results: accuracies improve considerable if the number of stories is available, confirming results of Stiller et al. [11].
- Regarding the statistical method, we strongly advise against its use, since it leads to misunderstanding. Applying the two-fold validation strategy, we could identify that the validation of the statistical population using continuous spatial units (here L1) reports a better performance than dasymetric methods for the medium and high-resolution population maps, and reasonably good results in the very high-resolution approach. This has previously also been reported by Reed et al. [16] and Grippa et al. [42]. However, the validation using non-continuous spatial units as the urban blocks (here L0) revealed that this is a misperception. The accuracies are rather low with R^2^ around 30% and errors near to 100%, providing by far the worst accuracies among all experiments. Therefore, we discourage applying this method for the disaggregation of the population at any resolution level, even if the accuracies of the random forest models are robust. This could be related to a different underlying link between the population density and the covariates at the source spatial unit (L2) and the grid cells where the weights are calculated and used for the allocation of population.
- The combined method using the statistical weight and the urban mask for the dasymetric disaggregation is the one taking more computational effort and featuring higher complexity. However, for the MR and the HR scenarios the improvement of the population maps is important when data on land use, vegetation and topography are used (cases A and B). Employing the MR mask, the accuracy metrics improve up to 6% and the MAPE by 22%, indicating a lower presence of extreme outliers. Similarly, the HR masks improve up to 10%, also diminishing extreme outliers. In both cases, the overestimations are very similar, and the improvement is almost negligible. Considering the VHR scenarios, the weights created through the statistical models do not reflect the underlying condition of population density better than the building footprints themselves. As previously mentioned in (ii), only in one case the results are better than for the dasymetric ones; however, in the combined method there are two improvements, i.e., the extreme outliers and overestimations are lower. Nevertheless, the other accuracy statistics show that it is not worth the efforts since the additional effort to calculate the weight for the disaggregation is futile. Therefore, combined methods are recommended when medium- and high-resolution remote sensing data and enough ancillary geospatial information, such as land use, vegetation, topography, road networks, etc. are available, supporting the fine spatial allocation of population.
- Regarding the categorical disaggregation, we found that the weights based on the land use are able to successfully distribute more population to denser populated units. We compared different occupancy rates at two levels to minimize errors and demonstrated that when L0 population data is missing, other available validation data can be used to calculate the occupancy rates with similar outcomes; however, the effect of the weights is not always strong enough to distribute population properly within land uses when the diversity is not fully captured by the land use map. This is clearly related to the estimated weights for commercial and other land uses, even if the selection of the weight was done to minimize errors. The weights provided better results in residential and mixed land use spatial units, but units with commercial or other land uses as primary use add many uncertainties. In this sense, along with imprecise land use maps, errors in census data (i.e., unpopulated urban blocks with residential buildings and highly populated blocks with non-residential buildings) affect the optimal selection of weights for the categorical methods. However, this effect cannot be quantified due to the inexistence of data.
- The use of several accuracy metrics allowed for a more complete picture of the performance of the models. Based on our experimental outcomes, we recommend the use of either R^2^ or RMSE, since both provide a similar response when comparing the experiments, the RTAE to report a global error subject to total population, and the MAPE to capture the extreme outliers, reflecting the proportional error of the spatial units. This metric cannot be calculated if there are non-populated units, i.e., these units should be discarded in its computation. The overestimation and underestimation errors are required when non-contiguous validation data are used, since the other metrics cannot quantify population errors outside the spatial units. Consequently, our advice is to at least use three relative accuracy metrics (e.g., RMSE, RTAE and MAPE) for a not one-sided estimation of accuracies, since absolute errors are less suitable for comparison with other studies due to its high dependency on the level, size, and density of the validation units.

In order to interpret these general findings, however, we should keep the following limitations in mind: On the one hand, we based the experiments only on a single city. Although Medellin is a good exemplary case because of its urban, socioeconomic and topographical diversity, we are aware that every city is unique, and Medellin is not a blueprint for any city across the globe. Although we expect similar outcomes in different cities, it still needs to be empirically proven in other regions. On the other hand, we used a comprehensive set of covariates; however, the transferability of the statistical model between administrative units and the 100m-grid is not straightforward, which means that we were not able to find the statistical relationship between population density and spatial variables across different sized spatial units. In this sense, a sensitivity analysis of transferability with varying spatial units is suggested.

Another consideration is that there are significant differences in the errors based on the size, population density and land use of the spatial unit. This corresponds with previous studies that also found relationships between higher errors and lower percentages of residential land use and higher mixed or other land uses [11] and population density [21]. Therefore, the physical configuration of an urban landscape affects the performance of the disaggregation methods. Hence, highly heterogeneous cities, such as is our case in Medellin, might have higher errors and extreme outliers. In turn, we expect for cities with more homogeneous landscapes lower errors. In this regard, in an attempt to improve the accuracy of results there are some considerations for future research. First, the addition of image texture metrics in the combined method to add information on spatial heterogeneity in the models [61] is worth exploring. Second, minimizing misclassification in remotely sensed-derived data. For example, removing road infrastructure from the urban masks would reduce population overestimation in unpopulated areas. Third, the land use map is indicating the main land use purpose, but the veracity at the building level is unknown. Adjusting the land use data with ancillary data such as establishments in this study or points of interest from Open Street Maps, could improve the calculation of occupancy rates and thus the categorical approach [62]. And fourth, by testing and including new covariates that provide relevant information on the distribution of population in different urban configurations and better explain their statistical relationships independently of the spatial unit.

Regarding the data used in this study, we are aware that the best performing method relies on VHR geodata, in this study building footprints from cadastre. Unfortunately, these data are not available in every context. For this reason, in the future, the use of alternative VHR datasets is worth exploring. Currently there are potential datasets that might overcome their lack. For instance, some authors used land use, building footprints, road networks, and points of interest data from OpenStreetMap to disaggregate population onto buildings [63] and grids [64]. Other promising datasets are the openly available Google and Microsoft building footprint created by means of artificial intelligence and aerial imagery that cover many regions in the world [65, 66]. The emergence of these VHR datasets is an important milestone for population mapping in the Global South. Nonetheless, data from remote sensing are still very valuable for many applications and particular context: countries where VHR data are uncompleted, for multitemporal population mapping applications [51], to produce up-to-date population mapping in fast-growing and/or informally developed urban areas, as well as for national- /global-wide population mapping [26–29].

Ultimately, another core contribution of this study is the production of the best population grid using the population counts from the census urban blocks (L0) and the determination of the best performing method: the categorical dasymetric method using the building heights and land use. These highly detailed population maps at the building level as well as at the 100x100m-cell grid level are not commonly existent in the Global South. Such products at this spatial resolution and with high accuracy have a great applicability in several fields, for example in risk assessment [67] or in urban planning [10]. And it can also be the basis for transferring the approach to other Colombian cities with available cadastre data.

## Conclusions

We conducted several experiments on the performance of various population disaggregation methods. We based them on different scenarios of data availability with the aim to provide recommendations to researches that attempt to produce fine-grained population maps using remote sensing and geospatial information. In total, five top-down methods were described and tested on medium, high and very high-resolution data. Besides, we applied a two-fold validation approach that allowed finding misperceptions when evaluating the methods and use several accuracy metrics that helped to identify differences in the quantified errors and extreme outliers.

Based on our results, we provide a list of recommendations that can be used as guideline, in general but especially in dense and heterogeneous urban landscapes, to support those interested in disaggregating population from coarse spatial units to fine-grained population distribution maps according to their objective and depending on the amount and level of detail of the input data. We suggest the combination of statistical and dasymetric methods when remotely sensed data and at least topography and road network covariates are available, otherwise, the simple dasymetric method can be used with certain limitations. On the contrary, we highly recommend dasymetric methods when data on building footprints are available, since they produce a reliable distribution of population. With respect to the validation we encourage the use of at least three relative accuracy metrics, since their values depend on the validation level and method. These recommendations are thought to save additional efforts, time and ease comparison with other population products in future population mapping. In conclusion, one unsurprising result is: the better the data and the higher the resolution of the geodata, the more accurate the resulting population map. However, a central result is that we found the combination of statistic and dasymetric methods to provide better results when only remotely sense data is available. On the contrary, the use of statistical methods is not recommended due to the false perception of higher accuracy depending on the evaluation level.

This research contributes to the field of high-resolution population mapping suggesting for specific data availability scenarios the most efficient method. This is of particular relevance in the face of developing and implementing tailored actions in several fields that require the knowledge of populated areas.

## Supporting information

S1 Fig Iterative test experiment to determine the most suitable combination of occupancy proportions for commercial and other land uses at L0. Where the occupancy of residential buildings is 100% and the values of commercial (C) and others (O), respectively, are shown in the X-axis (C%_O%). The normalized root mean square error (RMSE), relative total absolute error (RTAE), underestimation and overestimation are measured for each pair combination for the VHR (grey) and 3D VHR (blue) urban masks. The weight 40_30 minimizes errors between estimated and reference population based on the four-accuracy metrics for both the area and building size urban masks.
(TIF)

S2 Fig Iterative test experiment to determine the most suitable combination of occupancy proportions for commercial and other land uses at L1 and comparison with L0. Where the occupancy of residential buildings is 100% and the values of commercial (C) and others (O), respectively, are shown in the X-axis (C%_O%). The normalized root mean square error (RMSE) and relative total absolute error (RTAE) are measured for each pair combination for the VHR (grey) and 3D VHR (blue) urban masks. The weight 40_30 minimizes errors at L0, it is compared to all combinations of occupancy rates that minimize errors at L1 and L0 for both the area and building size urban masks. Occupancy rates between 10% to 40% of commercial and other land uses provide the best results, presenting slight differences in the accuracy metrics.
(TIF)

S3 Fig Violin plots with the distribution and density of absolute percentage errors (APE) grouped by quantiles using the area (m^2^) of the validation zones (VZ) by experiment, for the VZ L0 using the urban blocks. From left to right errors are shown from smaller (green) to larger units (pink), showing statistical differences between groups. The dot reports the median APE per area-based group, while the number between brackets in the x-axis reports its mean.
(TIF)

S4 Fig Violin plots with the distribution and density of absolute percentage errors (APE) grouped by the population density (P/ha) of the validation zones (VZ) by experiment, for the VZ L0 using the urban blocks. From left to right errors are shown from less (yellow) to most densely (blue) populated units, showing statistical differences between groups. The dot reports the median APE per area-based group, while the number between brackets in the x-axis reports its mean.
(TIF)

S5 Fig Result of the best population grid (P_L0_). Using census population at L0 as source zones and the categorical dasymetric method with 3D VHR and land use data. The best population grid map for the city of Medellin is also available in shapefile format at: https://doi.org/10.6084/m9.figshare.c.5857320.v1.
(TIF)
