# Supplementary figures and images for: Empiric recommendations for population disaggregation under different data scenarios
Source: PLoS One. 2022 Sep 16;17(9):e0274504. doi: 10.1371/journal.pone.0274504 (PMC9481046; doi:10.1371/journal.pone.0274504)

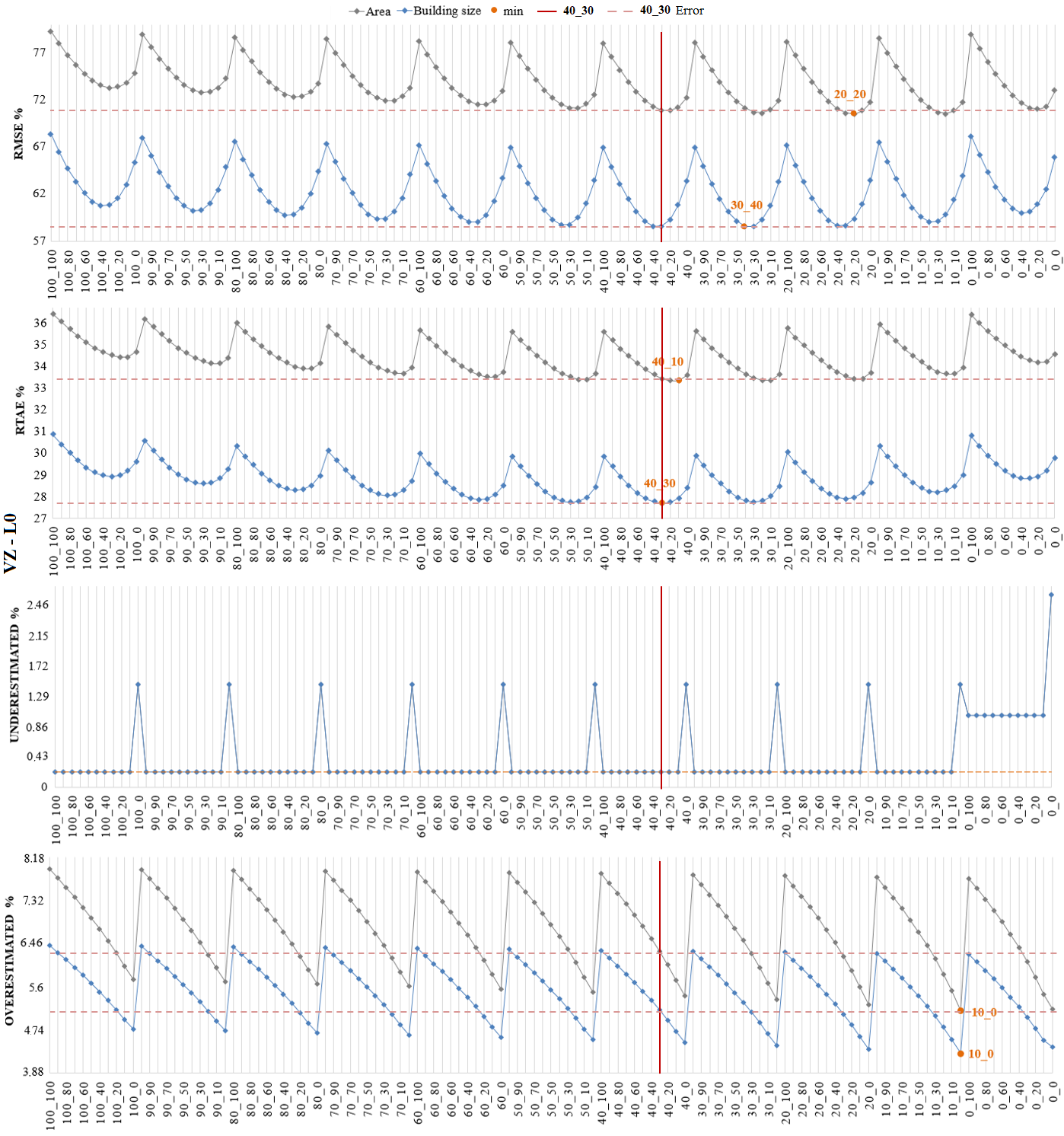

Supplement: S1 Fig — Where the occupancy of residential buildings is 100% and the values of commercial (C) and others (O), respectively, are shown in the X-axis (C%_O%). The normalized root mean square error (RMSE), relative total absolute error (RTAE), underestimation and overestimation are measured for each pair combination for the VHR (grey) and 3D VHR (blue) urban masks. The weight 40_30 minimizes errors between estimated and reference population based on the four-accuracy metrics for both the area and building size urban masks. (TIF) [file pone.0274504.s001.tif]

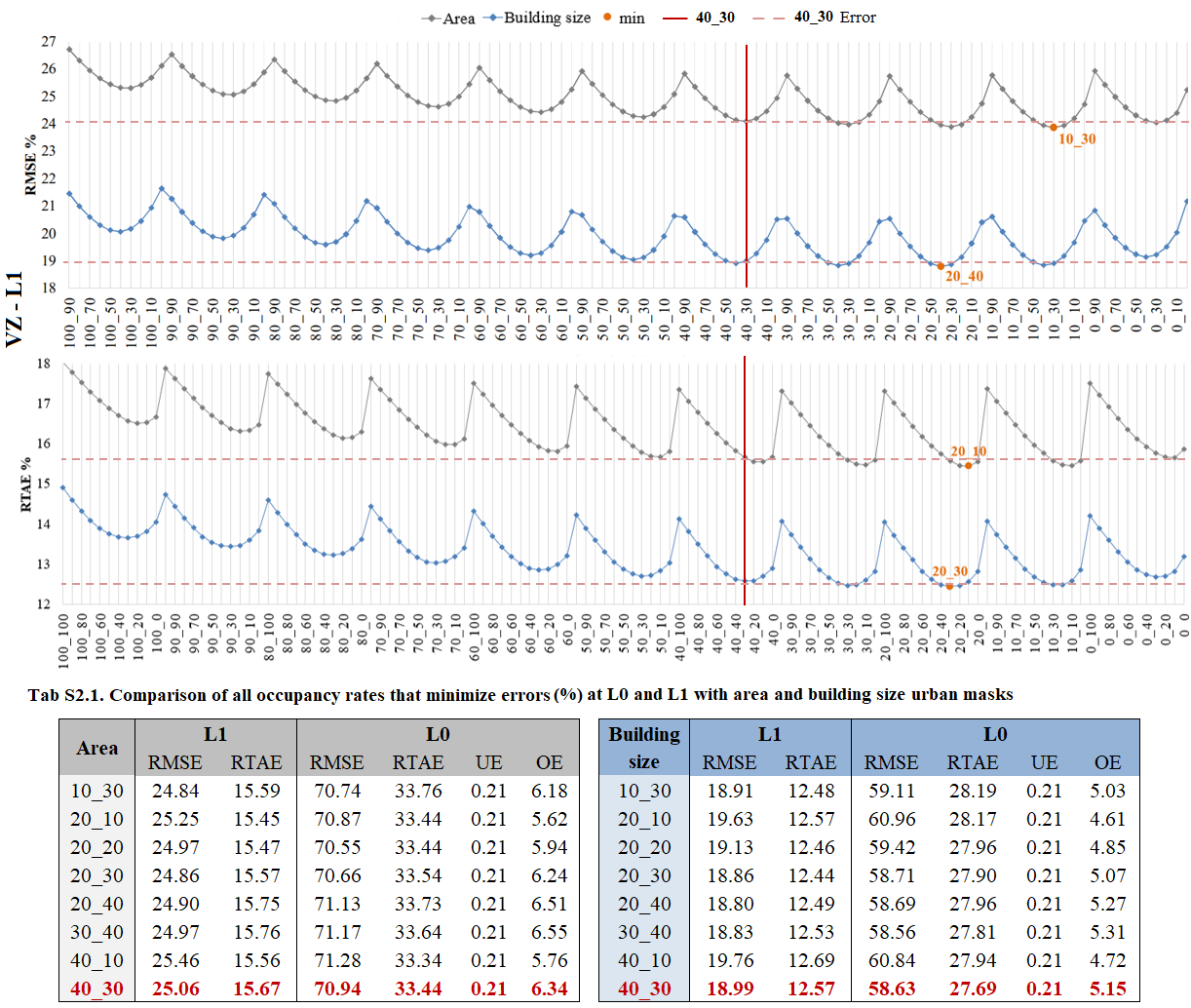

Supplement: S2 Fig — Where the occupancy of residential buildings is 100% and the values of commercial (C) and others (O), respectively, are shown in the X-axis (C%_O%). The normalized root mean square error (RMSE) and relative total absolute error (RTAE) are measured for each pair combination for the VHR (grey) and 3D VHR (blue) urban masks. The weight 40_30 minimizes errors at L0, it is compared to all combinations of occupancy rates that minimize errors at L1 and L0 for both the area and building size urban masks. Occupancy rates between 10% to 40% of commercial and other land uses provide the best results, presenting slight differences in the accuracy metrics. (TIF) [file pone.0274504.s002.tif]

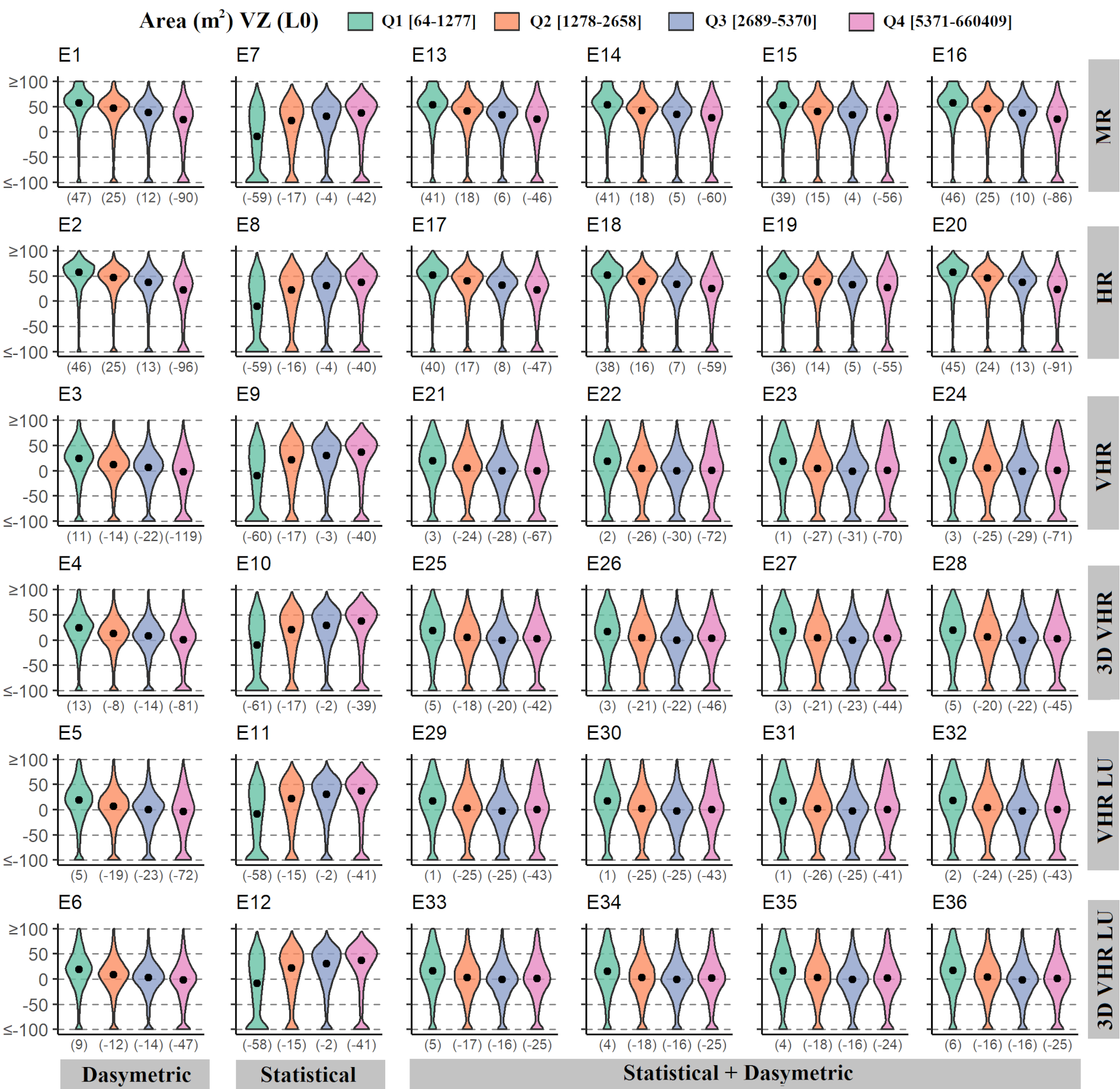

Supplement: S3 Fig — From left to right errors are shown from smaller (green) to larger units (pink), showing statistical differences between groups. The dot reports the median APE per area-based group, while the number between brackets in the x-axis reports its mean. (TIF) [file pone.0274504.s003.tif]

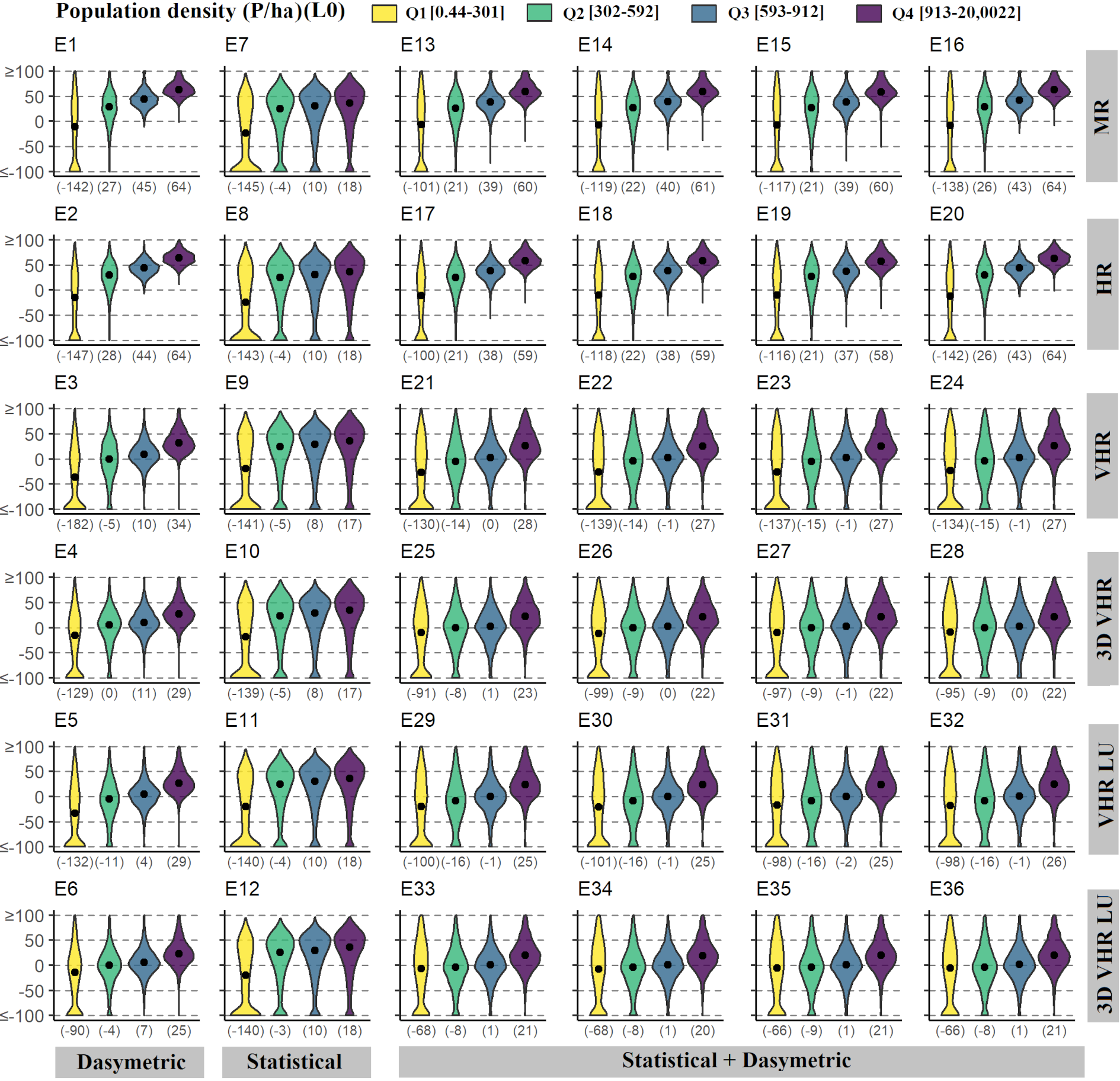

Supplement: S4 Fig — From left to right errors are shown from less (yellow) to most densely (blue) populated units, showing statistical differences between groups. The dot reports the median APE per area-based group, while the number between brackets in the x-axis reports its mean. (TIF) [file pone.0274504.s004.tif]

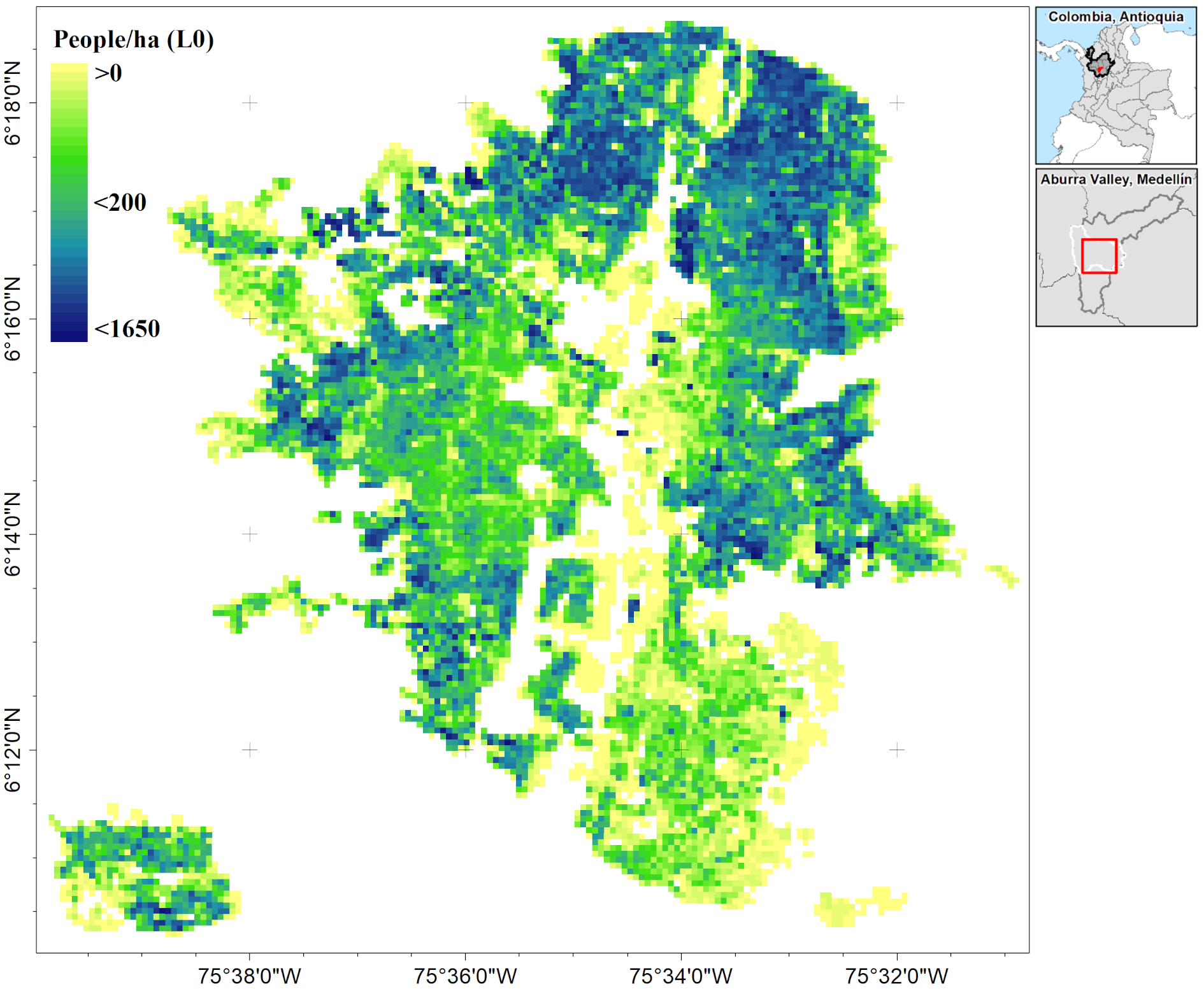

Supplement: S5 Fig — Using census population at L0 as source zones and the categorical dasymetric method with 3D VHR and land use data. The best population grid map for the city of Medellin is also available in shapefile format at: https://doi.org/10.6084/m9.figshare.c.5857320.v1. (TIF) [file pone.0274504.s005.tif]
